# A Prostate Cancer Model Build by a Novel SVM-ID3 Hybrid Feature Selection Method Using Both Genotyping and Phenotype Data from dbGaP

**DOI:** 10.1371/journal.pone.0091404

**Published:** 2014-03-20

**Authors:** Sait Can Yücebaş, Yeşim Aydın Son

**Affiliations:** 1 Medical Informatics Department, Graduate School of Informatics, Middle East Technical University. Ankara, Turkey; 2 Bioinformatics Graduate Program, Graduate School of Informatics, Middle East Technical University, Ankara, Turkey; Eberhard-Karls University, Germany

## Abstract

Through Genome Wide Association Studies (GWAS) many Single Nucleotide Polymorphism (SNP)-complex disease relations can be investigated. The output of GWAS can be high in amount and high dimensional, also relations between SNPs, phenotypes and diseases are most likely to be nonlinear. In order to handle high volume-high dimensional data and to be able to find the nonlinear relations we have utilized data mining approaches and a hybrid feature selection model of support vector machine and decision tree has been designed. The designed model is tested on prostate cancer data and for the first time combined genotype and phenotype information is used to increase the diagnostic performance. We were able to select phenotypic features such as ethnicity and body mass index, and SNPs those map to specific genes such as *CRR9*, *TERT*. The performance results of the proposed hybrid model, on prostate cancer dataset, with 90.92% of sensitivity and 0.91 of area under ROC curve, shows the potential of the approach for prediction and early detection of the prostate cancer.

## Introduction

In Genome Wide Association Studies (GWAS) Single Nucleotide Polymorphisms (SNP)-complex disease associations are searched such as, age related macular degeneration [1], heart diseases [2], diabetes [3], rheumatoid arthritis [4], Crohn's Disease [5], Hypertension [6], Multiple Sclerosis [7] and cancer types [8]–[9]–[10] neurodegenerative diseases [11] and psychiatric diseases such as bipolar disorder [12]. Current GWAS of SNP profiles with such chronic and complex diseases are leading to the discovery of different genetic loci and individual SNPs related with the conditions, but association of only SNP genotyping profiles are not strong enough for prediction of disease condition. So, this study is designed to test the hypothesis if and to which degree integrating genotype profiles and the phenotypic features; including demographic information, environmental factors, life-style habits along with clinical findings of a patient will strengthen the predicative performance of the disease models. So far there isn't any publication that combines multiple genotypic and multiple phenotypic features, which would require implementation of new data mining approaches that can handle data with such different characteristics and even higher dimensionality.

Methods used in GWAS can be grouped under two main categories which are parametric and non-parametric [13]. Non-parametric methods do not require a genetic model given beforehand; instead they build their own models based on given data by using data mining and machine learning [13]. Non-parametric methods are preferred due to the high dimensionality of the genetic data in which traditional statistic methods are not sufficient enough for the analysis [14]. Almost all known machine learning algorithms have been used in GWAS, some of the foremost methods are Decision Trees [15]–[16], Artificial Neural Networks [16], Bayesian Belief Networks [17], Support Vector Machines [18]–[19]–[20] and Genetic Algorithms [21]. For the analysis of genotyping data, as observed from various applications of data mining, there is no clear evidence that any of the methods performs better than others [13]. All methods have their own advantages and disadvantages, and the selection of the appropriate method is mostly based on the given problem, data type, study design and aim of the work. There are also few examples for the application of different hybrid data mining approaches with GWAS data to increase the predicative performance, in which one main method is selected and genetic based algorithms, are used as the second step for the optimization of the main method [22].

Here, for first time we are introducing a hybrid feature selection model combining two non-parametric data mining methods, SVM and ID3, for the determination of most predictive phenotypic and genotypic features related with a complex disease. As distinct from many works in the literature, in this study we have used both methods individually rather than just optimizing the main method. The prostate cancer data is used as a case study and we have demonstrated that combining genotype information with phenotypes has better predictive performance than using only genotypes or only phenotypes in disease diagnosis, while exceeding the performance of prostate specific antigen (PSA) screening test [23].

## Materials and Methods

### Prostate Cancer Data Set

The dataset, “Multi Ethnic Genome Wide Scan of Prostate Cancer”, used in this work is downloaded from NCBI's dbGaP database and has an accession number phs000306 version 2. This data consists of 4650 cases and 4795 controls with three different ethnicities, African Americans, Latinos and Japanese. Each individual in the study has 600,000 SNPs and 20 phenotypes and the number of subjects that contains both phenotypic and genotypic attributes is 9130.

### Data Preprocessing

Data preprocessing consisted of three steps. In the first step Plink analysis was conducted in order to find the statistical power of relations between the genotype and the given disease. The threshold for the association of the SNPs with prostate cancer was determined as p<0.005 after the GWAS and 22,848 SNPs satisfying this condition formed the first representative subset. At second step METU-SNP's AHP (Analytical Hierarchical Process) feature was used to prioritize SNPs based on the biological and the statistical significance, which filtered the associated SNPs down to 2710 SNPs.

Data matching, cleaning and transformation were done in the final step of the data preprocessing. The genotypic and the phenotypic attributes of the subjects are combined in the data matching step based on the subject ID's and the subject ID conversions given in the manifest data. In the cleaning phase missing values caused by phenotypic attributes were replaced by class mean calculation and the attribute was deleted where class mean cannot be calculated. Data transformation was needed to code the alleles because SVMs use numerical values instead of categorical ones. In literature allele combinations are coded by three numeric values based on the heterozygous and homozygous major alleles [18]. Disadvantage of these schemes are that “*the alleles are not treated symmetrically* [*18*]”. As the parent of origin was not indicated in our data we used an alternative coding scheme, in which symmetric alleles are treated in the same manner. This coding scheme is presented in Table 1.

**Table 1:** Major allele coding scheme.

Major Alleles	Coding Value
AA	1
AT/TA	2
AC/CA	3
AG/GA	4
TT	5
CT/TC	6
GT/TG	7
CC	8
GC/CG	9
GG	10

### Analysis

According to the literature the most widely used algorithms for detecting the relations between genotype information and the disease are ANN, SVM and Decision Trees. There are also examples for applications of different data mining approaches in a hybrid manner to increase the predicative performance where one main method is selected and genetic based algorithms are used as the second step for optimization of the main method [15]–[22].

In our model we've combined two different methods, SVM and ID3, and for each of these methods an appropriate optimization was applied rather than combining a main method with an advanced optimization as stated above. By this way instead of benefitting from one strong method, we've combined the strengths of different methodologies; ID3's robustness to noise and outliers [24] as well as its power to handle non-linear problems and SVM's prediction performance over non-linear binary classification problems. Also both methods are more interpretable when compared to other methods.

Our SVM-ID3 Hybrid Model was constructed in RapidMiner 5.0 which is a free open source software tool for data mining applications and preferred in various applications in the literature such as [25]. For the SVM phase RBF kernel is chosen. This kernel is widely used in GWAS [19] and preferred in our study for its faster learning speed and its advantage of to be used as both linear kernel and sigmoid kernel in some special conditions [26]. Besides the kernel function SVM has two important parameters (C,γ) if not adjusted well, could cause overfitting or underfitting of the condition. The ***C*** constant is used to adjust the margin of the hyperplane that separates the classes and gamma parameter gives its shape to decision boundary. Optimization of these parameters has been reported previously [27], and we have selected to apply the grid search approach for the optimization, which has been described previously [28]. The value ranges for C and gamma, used during the grid search is decided based on literature [27] along with our own experience with the data. For gamma the value range is selected in between [0.0001, 100] with powers of ten and the value range for C is selected in between [0–10] with five linear steps. The grid search for SVM optimization has lasted around ten hours to complete in a system with a 16 GB memory and 3.4 GHz Intel Core i7 processor, revealing 42 combinations.

In literature there are various studies that combine SVMs and decision trees. Although previously published hybrid models of SVM and decision trees (SVM-DT) are generally used for multi-classification and multi-clustering problems, there are also examples of the SVM-DT combinations used for binary classification problems [29]. In all of the cases the SVM-DT models, SVM is applied first in order to optimize the parameters and the datasets to be used next in the decision tree. In our study we have also applied SVM in the first step, however instead of ranking the attributes and selecting the top listed ones according to SVM weights, which present a risk for loss of information, we have used the entire SVM weights as the weight feature in ID3. These weights for the ID3 attributes are calculated according to the formula given below.
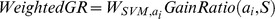
The ID3 Tree is implemented on RapidMiner with weighting strategy explained above. A second grid search was run in order to find the optimum value for weighted information gain ratio. The range for this value was set in the range [10^−3^, 10] and searched by 50 logarithmic steps which resulted in 51 combinations and completed in 11 hours.

**Figure 1:**
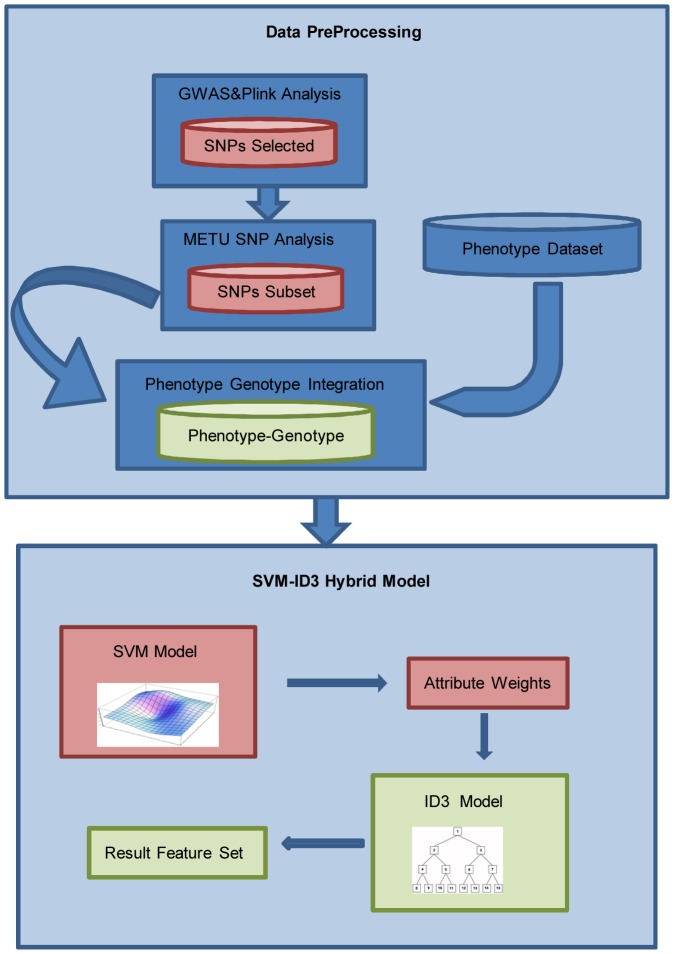
Overall Work flow of the SVM-Tree Hybrid Model. Overall workflow starts with data preprocessing where representative SNP subset is formed by Plink and METU-SNP analysis, phenotype and genotyping data integrated and missing values are either eliminated or manually filled by class mean calculation. After the data preprocessing, integrated dataset is fed into hybrid model where SVM model gives the attribute weights which are used in ID3.

The overall workflow for the data pre-processing, which also includes GWAS and integration of phenotype and genotyping data, and the Hybrid SVM-Tree model described here is summarized in Figure 1.

## Results

In the first phase only SVM model was run to present the classification performance of the stand-alone method on three different datasets. First and the second set was either only genotyping or phenotype data and the third dataset contained both genotyping and phenotype data. The results of the standalone SVM model are given in Table 2.

**Table 2:** Performance comparison of stand-alone SVM model.

Performance Criteria	Only-Genotype Dataset	Only-Phenotype Dataset	Integrated Genotype and Phenotype Dataset
Accuracy	59.02	68.23	**72.46**
Precision	61.29	76.80	**82.68**
Recall	63.15	70.12	**71.34**
AUC	0.606	0.768	**0.829**

SVM model is tested by three different datasets, only genotype, only phenotype and integrated phenotype and genotype sets. Integrated data set performs best among others in terms of the performance criteria given.

These results in the Table 2 clearly shows that combining phenotypic information with genotype data slightly increased the decision performance in all aspects of accuracy, precision, recall and AUC. The hybrid SVM-ID3 model is then applied on the same three datasets and the performance comparison is presented in the Table 3.

**Table 3:** Performance comparison of SVM-ID3 Hybrid Model.

Performance Criteria	Only-Genotype Dataset	Only-Phenotype Dataset	Integrated Genotype and Phenotype Dataset
Accuracy	71.67	84.23	**93.81**
Precision	72.69	86.20	**96.55**
Recall	68.96	83.78	**90.92**
AUC	0.674	0.857	**0.91**

The hybrid SVM-ID3 model is tested on the same datasets, only genotype, only phenotype and integrated phenotype and genotype sets. Integrated data set performs best among others in terms of the performance criteria given.

**Figure 2:**
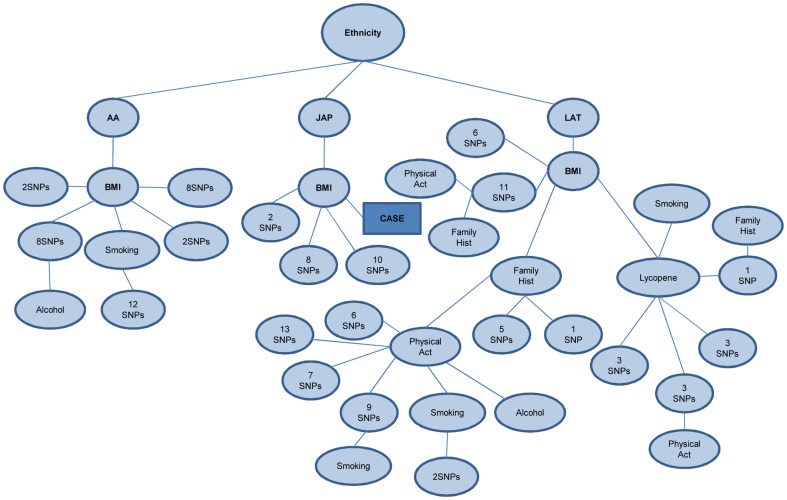
Overall tree structure of the hybrid model. The main tree is given in the Tree S1 material because the structure is too big. This figure is a small representation of main tree. Decision starts with ethnicity and African Americans are represented by AA, Japanese by JAP and Latinos by LAT. For all ethnicities the most descriptive phenotypic attribute is body mass index (BMI). Other phenotypic attributes that are in upper levels of tree are smoking behavior, family history, lycopene intake and physical activity. The number of SNPs in the nodes indicates the total number of SNPs found in different levels on that particular path of the tree.

According to SVM ID3 hybrid model structure, given in Tree S1, the most important attribute is the ethnicity. Our model made a strict distinction on ethnicity attribute, which leads different decision paths for African American, Latino and Japanese subjects. For all ethnicities the body mass index (BMI) attribute is the second descriptive feature of the decision path. For African American population descriptive phenotypes on different levels of tree are the attributes that indicate smoking and alcohol consumption habits. Surprisingly only phenotypic attribute found for Japanese population is the BMI. Attributes indicating family history, physical activity, lycopene intake and smoking behavior are observed for Latin population. The overall tree structure of the hybrid model is presented in the Figure 2.

Some of the prominent decision paths extracted from tree are mainly based on ethnicity. For example if the subject's ethnicity is African American and its BMI is in first category, which is BMI<22.5, by looking at rsid 11729739 our hybrid system can decide whether the subject is a case or control. If the allelic profile for this SNP is TT then the subject is called as a case, but if the subject is heterozygous carrying CT, than the subject is called as a control. When the results of hybrid system for Japanese population are examined, the BMI was also in the first level of decision path. If the subjects are in fourth branch of BMI, which is > = 30, then these subjects are directly classified as case. If the subjects are in first branch of BMI then the decision is made based on the SNP rs2442602; the subjects homozygous for the major allele (with AA genotype) are called as cases, but the decisions for the subjects carrying other alleles require investigation of additional SNPs.

The tree structure shows that the decision path for Latin population is more complex than the Japanese or African American populations. If the subjects are in first category of BMI then the subjects heterozygous for SNP rs17799219, carrying AG, are called healthy. If the subjects are in third category of BMI, which is <29.9, then a second phenotypic attribute, family history must be examined. If these subjects have first degree relatives with prostate cancer, then SNP rs6475584 is examined, to call if the subject is a case or not. Many rules, like given above, can be extracted from tree structure given in the Tree S1.

Overall our hybrid model identified 28 SNPs for African American, 22 SNPs for Japanese and 65 SNPs for Latino populations. We have investigated the SNPs mapping to genes within the SNPNexus database [30] and the non-coding SNPs through RegulomeDB [31] in order to see if they have been associated with prostate cancer or any other condition before.

When the SNPs found by hybrid model are searched through SNPnexus, 107 unique rsIDs matched with 62 unique Entrez GeneID and 42 of them were previously found to be associated with a condition listed in Genetic Association of Complex Diseases and Disorders (GAD) database. A representative set of genes- phenotypes and disease classes is given in the Table 4 and the whole list can be found in Table S1 material.

**Table 4:** SNPnexus results.

Gene	Entrez gene	Phenotype	Disease Class	Pubmed
MCPH1	79648	Adenocarcinoma|Pancreatic Neoplasms	CANCER	19690177
MCPH1	79648	breast cancer	CANCER	20508983
SMARCA4	6597	breast cancer	CANCER	19183483
CSMD1	64478	Chromosomal Instability|Cystadenocarcinoma, Serous|Ovarian Neoplasms	CANCER	19383911
CSMD1	64478	Chromosomal Instability|Cystadenocarcinoma, Serous|Ovarian Neoplasms	CANCER	19383911
MTAP	4507	Melanoma|Nevus|Precancerous Conditions|Skin Neoplasms	CANCER	19578365
MTAP	4507	melanoma|Nevus|Skin Neoplasms	CANCER	20574843
MTAP	4507	melanoma|Nevus|Skin Neoplasms|Sunburn	CANCER	20647408
MTAP	4507	Precursor Cell Lymphoblastic Leukemia-Lymphoma	CANCER	19665068
ST6GALNAC3	256435	Alcoholism	CHEMDEPENDENCY	20421487
ANGPT2	285	BMI- Edema rosiglitazone or pioglitazone	PHARMACOGENOMIC	18996102
KLF7	8609	Body Weight|Diabetes Mellitus, Type 2|Obesity|Overweight	METABOLIC	19147600
MTAP	4507	diabetes, type 2	METABOLIC	11985785
PACRG	135138	male infertility	REPRODUCTION	19268936
SEMA5B	54437	Tobacco Use Disorder	CHEMDEPENDENCY	20379614

The SNPs found by hybrid system are searched through SNPnexus. Many of them are found to be associated with specific genes and phenotypes. This table lists some of the genes that are matched by SNPs. As the disease class and phenotype indicates, our findings match with cancer disease class and the phenotypes searched for prostate cancer such as body mass index, smoking and drinking habits.

The non-coding SNPs in our final disease model are investigated through RegulomeDB, which showed that the SNPs found by our hybrid model have regulative effects. Table 5 below shows the SNPs with score lower than 4 from RegulomeDB. The whole list is given in the Table S2 material.

**Table 5:** High score SNPs from RegulomeDB.

rsID	Hits	score
rs1433369	Motifs|Footprinting|IRF, Motifs|PWM|DMRT5, Motifs|Footprinting|DMRT5, Motifs|Footprinting|STAT1, Motifs|PWM|IRF, Motifs|PWM|STAT1, Chromatin_Structure|FAIRE, Chromatin_Structure|DNase-seq, Protein_Binding|ChIP-seq|SMARCB1, Protein_Binding|ChIP-seq|POLR2A	2,2
rs11790106	Motifs|Footprinting|Pax-6, Motifs|PWM|Pax-6, Chromatin_Structure|FAIRE, Chromatin_Structure|DNase-seq, Protein_Binding|ChIP-seq|GATA1, Protein_Binding|ChIP-seq|HNF4A, Protein_Binding|ChIP-seq|HEY1, Protein_Binding|ChIP-seq|EP300, Protein_Binding|ChIP-seq|SMARCC2, Protein_Binding|ChIP-seq|CEBPB, Protein_Binding|ChIP-seq|FOXA2, Protein_Binding|ChIP-seq|NR3C1, Protein_Binding|ChIP-seq|STAT3, Protein_Binding|ChIP-seq|POLR2A, Protein_Binding|ChIP-seq|FOXA1, Protein_Binding|ChIP-seq|SRF, Protein_Binding|ChIP-seq|CDX2	2,2
rs6774902	Motifs|PWM|MAF, Motifs|PWM|c-Ets-1, Motifs|Footprinting|c-Ets-1, Motifs|Footprinting|MAF, Chromatin_Structure|DNase-seq, Protein_Binding|ChIP-seq|RAD21, Protein_Binding|ChIP-seq|CTCF	2,2
rs17701543	Motifs|PWM|CP2, Chromatin_Structure|DNase-seq, Protein_Binding|ChIP-seq|CTCF	3,1
rs12644498	Motifs|PWM|REST, Chromatin_Structure|FAIRE, Chromatin_Structure|DNase-seq, Protein_Binding|ChIP-seq|USF1	3,1
rs17375010	Chromatin_Structure|DNase-seq, Protein_Binding|ChIP-seq|CTCF	4
rs10788555	Chromatin_Structure|FAIRE, Chromatin_Structure|DNase-seq, Protein_Binding|ChIP-seq|STAT1, Protein_Binding|ChIP-seq|STAT3	4
rs6887293	Chromatin_Structure|FAIRE, Chromatin_Structure|DNase-seq, Protein_Binding|ChIP-seq|FOXA1, Protein_Binding|ChIP-seq|GATA3	4
rs744346	Chromatin_Structure|FAIRE, Chromatin_Structure|DNase-seq, Protein_Binding|ChIP-seq|ELK4	4
rs4562278	Chromatin_Structure|FAIRE, Chromatin_Structure|DNase-seq, Protein_Binding|ChIP-seq|HNF4A	4

The SNPs found by hybrid system are searched thorough regulomeDB. Many of the found to be affect binding and this table lists the SNPs with score lower than 4.

## Discussion

Here, we have presented a diagnostic disease model utilizing data mining methods, based on phenotype and genotyping data for the prostate cancer. Overall our results showed that the hybrid model developed by integrating SVM and ID3 methods is capable of using both genotype and phenotype information as input, and has the best performance for predicting the case vs. controls.

SVM is selected as the first step in our hybrid model as it is known for its high performance in GWAS [26], and ability to classify non-separable problems. The decision logic behind ANNs, which can also be utilized for GWAS, is not very clear because of its black box structure. Also ANNs have many parameters to adjust such as number of layers, number of nodes in layers, number of epochs and learning rate, and most importantly ANNs have the disadvantage of getting stuck at local minima. On the other hand SVMs has clear decision logic [20], has less number of parameters and due to the quadratic problem structure it only offers one solution, which is present at the global minima. As the second step in our hybrid model, ID3 decision tree is selected for its strong performance on classifying the discrete valued datasets as in GWAS. ID3 is easy to construct and works with good performance on noisy data with missing values, and easy to interpret with its visual features [24]. ID3 is also advantageous over C4.5 and CART trees because these methods construct trees by pruning which would hide some decision paths for the disease, and ID3 is also more suitable for categorical data.

To the best of our knowledge, there is no similar hybrid or stand-alone data mining method established as a gold standard for early diagnosis of prostate cancer. So, the performance results of the hybrid model had to be compared to the stand-alone SVM and ID3 models. The proposed Hybrid Model had better classification power over the stand-alone SVM and the ID3 model with all three datasets, where either only genotyping or phenotype data is used and for the integrated genotype-phenotype dataset. In the integrated genotyping-phenotype dataset the hybrid SVM-ID3 model with 90.92% sensitivity and 0.910 AUC outperformed the stand-alone SVM, and stand-alone decision tree which have 71.34% sensitivity and 0.829 AUC and 81.33% sensitivity and 0.732 AUC respectively. Additionally a three layer feed forward back propagation ANN structure was built in Rapid Miner and ran on the same combined genotype-phenotype dataset for comparison of performances. The execution run for 3 days to complete and the performance results in terms of accuracy, precision, and recall was all under 55%. Performance of ANN could be increased by optimizing the parameters used but this would cause the execution time to increase even higher. Even if the ANN could reach the same performance as the hybrid model, the long execution time would stand as another big disadvantage besides it being a black box algorithm.

Overall, our hybrid model was capable of efficiently using the high-volume, high-dimensional integrated genotyping and phenotype data as input. Currently, there are many published studies focused on analysis of genotyping data, but no example of combining phenotype with genotyping profile has been presented yet. Infilling this gap, for the first time genotyping and phenotype data are integrated together to build a diagnostic disease model for prostate cancer. As we have presented in Table 3, integrating the phenotype and genotype data increased the decision performance by terms of sensitivity and AUC. Sensitivity of the proposed hybrid model on a dataset with only genotypes is 68.69%, with only phenotypes is 83.78% where sensitivity increases to 90.92% when genotyping is integrated with phenotype data. In parallel to the sensitivity AUC value also increases; AUC for only genotyping data and only phenotype data are 0.674 and 0.857, respectively, but when both data is used AUC increases to 0.910.

In addition to its better classification performance, our results showed that the proposed SVM – ID3 Hybrid model was also able to identify the functional and regulatory SNPs related with prostate cancer. The selected SNPs and their gene-disease relations are checked by using the databases such as SNPnexus and RegulomeDB, which integrates third party information from different databases and studies in SNP-centric format. This means that the SNPs selected to build the diagnostic disease model with the proposed hybrid method are also candidates for further biological investigation of molecular etiology of the prostate cancer.

The proposed hybrid method has identified 107 unique SNPs for the diagnostic model out of 2710 highly associated SNPs selected after GWAS. When these 107 SNPs are searched in SNPnexus and RegulomeDB some of them are found to be related with specific genes and others affect regulation and binding. For example, rs2853668 is known to be associated with *CRR9, TERT* which plays an important role in the regulation of telomerase activity. The rs11790106 affects the regulation of *ATP2B2* gene which is important for energy production and calcium transportation of the cells. rs12644498 affects regulation of *ARL9* gene and rs6887293 affects the regulation of *AGBL4* which are also important for ATP/GTP cycle in cells. These genes are closely related to *IGF1* gene which plays an important role in insulin metabolism. Many of the genes, the 107 SNPs in the disease model map to, is related with growth and energy processes. These molecular functions are in fact related to the BMI, which the most important phenotypic attribute for all ethnicities found by our hybrid model.

Resulting feature set of our hybrid model was examined and phenotypic attribute ethnicity was found to be the most related attribute with the prostate cancer. This result was not surprising because several works in the literature already showed that there is a relation with ethnic features and prostate cancer disease. Kleinmann's work shows that the ethnic background of the patients plays an important role in the prostate cancer related quality of life [32]. According to Hoffman, the etiology of the prostate cancer is highly depended on ethnicity and African American's has the highest risk for having prostate cancer [33]. As a supporting result, our hybrid model strictly divides the prostate dataset according to ethnicity and for each ethnicity different paths were observed.

Although decision paths for ethnicities are all different, at the second level all decision paths indicate the BMI attribute. BMI is already known for its relations with different types of cancer such as breast cancer [34] and esophagus [35], and is also a strong phenotypic attribute for prostate cancer [36]. In literature along with BMI, age and family history, which are also among the selected attributes by our hybrid model, has been showed to be as important features for the diagnosis of the prostate cancer [36]. The preventive effect of high BMI values beyond 30 kg/m^2^ been stated previously [36], and interestingly for Japanese population we have also observed the same preventive effect of BMI for **morbid obese** cases at the lower levels of the decision path. Additionally, other most common phenotypic attributes in the decision paths such as family history, smoking habit, physical activity and lycopene intake were also associated with prostate cancer previously [37]. Overall, our results show that the proposed hybrid model included the previously established phenotypic attributes for prostate cancer.

Currently the blood Prostate Specific Antigen (PSA) levels is the gold standard for early detection of prostate cancer condition before biopsy, with the maximum sensitivity reported as 86%, and a specificity of 33% with AUC 0.67 [23]–[42]. PSA levels under 4 ng/ml is considered normal, levels between 4 ng/ml–10 ng/ml are known as suspicious and levels higher than 10 ng/ml known to be associated with high risk [38]. The problem with PSA test is determination of the thresholds. The range between 4 ng/ml–10 ng/ml is a grey area for decision and while some subjects below 4 ng/ml can have prostate cancer, but some above 10 ng/ml can still be healthy [39]. In addition, the cut off values also change with respect to the subject's age [40]. This introduces a serious problem and as the various literature state PSA should not be used as an early diagnosis tool in prostate cancer [41] until its performance is increased in terms of sensitivity and specificity [42]. When the diagnostic performance results of the proposed hybrid model with 90.92% sensitivity, and 0.91 AUC is considered, it presents a potentially good tool for the early detection of the prostate cancer. After validation with pilot studies, the proposed model which only requires a buccal swap would stand as a good alternative to blood PSA test.

Here, for first time we have proposed a predicative disease model integrating genotyping and phenotype data through a hybrid feature selection, which combines two non-parametric data mining methods, SVM and ID3. As distinct from many works in the literature, in this study we have used both methods individually rather than just optimizing the main method. The prostate cancer data is used as a case study and we have demonstrated that the model combining genotype information with phenotypes yields a better performance than using only genotype or phenotype data in disease diagnosis while also exceeding the performance of prostate specific antigen (PSA) screening test [23].

## Conclusions

In this study for the first time genotyping and phenotype data are integrated and a hybrid model of SVM-ID3 for prostate cancer is build. An important contribution of this work was the integration of genotyping with phenotype data. Effect of this integration is tested in both stand-alone SVM and SVM-ID3 hybrid model. In terms of performance measures such as sensitivity and AUC the integrated data set outperformed the datasets with only genotype and with only phenotype in both models. Sensitivity and AUC of integrated dataset for stand-alone SVM was 71.34% and 0.829 respectively. When the same integrated dataset is used in the hybrid model sensitivity increased to 90.92% and AUC increased to 0.91, also outperforming the blood PSA test. The model was able to identify prostate cancer associated SNPs that either map to a cancer specific genes such as *CRR9, TERT*, *ATP2B2*, *ARL9, and AGBL4* and/or with regulatory effects. Experimental and clinical validation of the described associations for prostate cancer can lead us to better understand the progression of the disease at the molecular level. Additionally, the descriptive phenotypes selected by the hybrid model were also previously identified features for their relations with prostate cancer in previous studies. Ethnicity was observed to be at the root of the decision tree structure, whereas BMI, family history and smoking were the other phenotypes that are at the top levels of the decision model. Overall, our study showed that the predictive disease model build with the hybrid SVM-ID3 approach based on genotyping and phenotype data provides a promising tool for early detection of the prostate cancer. After validation of the proposed model with pilot studies, it can be implemented as a clinical decision support module to evaluate patients risk to develop prostate cancer, and the phenotypes related to life style (BMI, exercise, smoking, etc..) that have high impact on patients risk can be identified for each individual to be monitored in the upcoming visits.

Further studies on the proposed hybrid SVM-ID3 method and other data mining approaches for the integrative analysis of the GWAS results and phenotypic information would aid in development of other successful disease models, which would excel the translation of variant-disease association findings into the clinical setting for the development of new decision support tools and personalized medicine approaches.

## Supporting Information

Table S1 **Whole list of SNPnexus results.**
(DOCX)

Table S2 **Whole list of RegulomeDB results.**
(DOCX)

Tree S1 **Text representation of tree structure.** The tree structure of the SVM-ID3 hybrid model.
(DOCX)

## References

[1] Klein RJ, Zeiss C, Chew EY, Tsai JY, Sackler RS, et al (2005) Complement Factor H Polymorphism in Age-Related Macular Degeneration. Science 308 (5720) 385–9 10.1126/science.1109557 15761122 PMC1512523

[2] Lettre G, Palmer CD, Young T, Ejebe KG, Allayee H, et al (2011) Genome Wide Association Study of Coronary Heart Disease and Its Risk Factors in 8,090 African Americans: The NHLBI Care Project. PLoS Genet 7 (2) e1001300 10.1371/journal.pgen.1001300 21347282 PMC3037413

[3] Reddy MV, Wang H, Liu S, Bode B, Reed JC, et al (2011) Association between type 1 diabetes and GWAS SNPs in the southeast US Caucasian population. Genes Immun 12 (3) 208–12 10.1038/gene.2010.70 21270831 PMC3952552

[4] Stahl EA, Raychaudhuri S, Remmers EF, Xie G, Eyre S, et al (2010) Genome-wide association study meta-analysis identifies seven new rheumatoid arthritis risk loci. Nat Genet 42 (6) 508–514 10.1038/ng.582 20453842 PMC4243840

[5] Lee JC, Parkes M (2011) Genome-wide association studies and Crohn's disease. Brief Funct Genomics 10 (2) 71–76 10.1093/bfgp/elr009 21436303

[6] Adeyemo A, Gerry N, Chen G, Herbert A, Doumatey A, et al (2009) A Genome-Wide Association Study of Hypertension and Blood Pressure in African Americans. PLoS Genet 5 (7) e1000564 10.1371/journal.pgen.1000564 19609347 PMC2702100

[7] Jakkula E, Leppä V, Sulonen AM, Varilo T, Kallio S, et al (2010) Genome-wide Association Study in a High-Risk Isolate for Multiple Sclerosis Reveals Associated Variants in STAT3 Gene. Am J Hum Genet 86 (2) 285–91 10.1016/j.ajhg.2010.01.017 20159113 PMC2820168

[8] Yeager M, Orr N, Hayes RB, Jacobs KB, Kraft P, et al (2007) Genome-wide association study of prostate cancer identifies a second risk locus at 8q24. Nat Genet 39 (5) 645–9. 17401363 10.1038/ng2022

[9] Easton DF, Eeles RA (2008) Genome-wide association studies in cancer. Hum. Mol. Genet 17 (R2) R109–R115 10.1093/hmg/ddn287 18852198

[10] Gerstenblith MR, Shi J, Landi MT (2010) Genome-wide association studies of pigmentation and skin cancer: a review and meta-analysis. Pigment Cell Melanoma Res 23 (5) 587–606 10.1111/j.1755-148X.2010.00730.x 20546537 PMC3179913

[11] Tsuji S (2010) Genetics of neurodegenerative diseases: insights from high-throughput resequencing. Hum. Mol. Genet 19 (R1) R65–R70 10.1093/hmg/ddq162 PMC2875051 20413655

[12] Scott LJ, Muglia P, Kong XQ, Guan W, Flickinger M, et al (2009) Genome-Wide Association and Meta-Analysis of Bipolar Disorder in Individuals of European Ancestry. Proc Natl Acad Sci U S A 106 (18) 7501–6 10.1073/pnas.0813386106 19416921 PMC2678639

[13] Musani SK, Shriner D, Liu N, Feng R, Coffey CS, et al (2007) Detection of Gene - Gene Interactions in Genome-Wide Association Studies of Human Population Data. Hum Hered 63 (2) 67–84. 17283436 10.1159/000099179

[14] Aguiar V, Seoane JA, Freire A, Guo L (2010) GA-Based Data Mining Applied to Genetic Data for the Diagnosis of Complex Diseases. In: MGestal Pose, DRivero Cebrián editors. Soft Computing Methods for Practical Environment Solutions: Techniques and Studies. Hershey: Information Science Reference. pp. 219–239. doi:10.4018/978-1-61520-893-7.ch014

[15] Huang J, Lin A, Narasimhan B, Quertermous T, Hsiung CA, et al (2004) Tree-structured supervised learning and the genetics of hypertension. Proc Natl Acad Sci U S A 20;101 (29) 10529–34. 10.1073/pnas.0403794101 PMC489971 15249660

[16] Anunciação O, Gomes BC, Vinga S, Gaspar J, Oliveira AL, et al.. (2010) A Data Mining Approach for the Detection of High-Risk Breast Cancer Groups. In: Rocha, M.P, Fernández Riverola, F, Shatkay, H, Corchado Rodríguez, J.M editors. Advances in Bioinformatics. Berlin Heidelberg: Springer. pp. 43–51.

[17] Mourad R, Sinoquet C, Leray P (2011) A hierarchical Bayesian network approach for linkage disequilibrium modelling and data-dimensionality reduction prior to genome-wide association studies. BMC Bioinformatics 12: 16 10.1186/1471-2105-12-16 21226914 PMC3033325

[18] Listgarten J, Damaraju S, Poulin B, Cook L, Dufour J, et al (2004) Predictive Models for Breast Cancer Susceptibility from Multiple Single Nucleotide Polymorphisms. Clin Cancer Res 10 (8) 2725–37. 15102677 10.1158/1078-0432.ccr-1115-03

[19] Huang LC, Hsu SY, Lin E (2009) A comparison of classification methods for predicting Chronic Fatigue Syndrome based on genetic data. J Transl Med 7: 81 10.1186/1479-5876-7-81 19772600 PMC2765429

[20] Abeel T, Helleputte T, Van de Peer Y, Dupont P, Saeys Y (2010) Robust biomarker identification for cancer diagnosis with ensemble feature selection methods. Bioinformatics 26 (3) 392–8 10.1093/bioinformatics/btp630 19942583

[21] Mooney MA, Wilmot B, McWeeney SK (2011) The GA and the GWAS: Using Genetic Algorithms to Search for Multi-locus Associations. IEEE/ACM Trans Comput Biol Bioinform 9 (3) 899–910 10.1109/TCBB.2011.145 22025762 PMC3748153

[22] Ritchie MD, Motsinger AA, Bush WS, Coffey CS, Moore JH (2007) Genetic programming neural networks: A powerful bioinformatics tool for human genetics. Appl Soft Comput 7 (1) 471–479. 20948988 10.1016/j.asoc.2006.01.013 PMC2952963

[23] Bretton PR, Evans WP, Borden JD, Castellanos RD (1994) The use of prostate specific antigen density to improve the sensitivity of prostate specific antigen in detecting prostate carcinoma. Cancer 74 (11) 2991–5. 7525040 10.1002/1097-0142(19941201)74:11<2991::aid-cncr2820741116>3.0.co;2-r

[24] Rokach L, Maimon O (2005) Decision trees. In: Lior Rokach, Oded Maimon, editors. Data Mining and Knowledge Discovery Handbook. Dordrecht, Heidelberg, London, New York: Springer. pp 165–187.

[25] Graczyk M, Lasota T, Trawiński B (2009) Comparative Analysis of Premises Valuation Models Using KEEL, RapidMiner, and WEKA. In: Ngoc Thanh Nguyen, Ryszard Kowalczyk, Shyi-Ming Chen, editors. Computational Collective Intelligence. Verlag Berlin, Heidelberg: Springer. pp 800–812. doi:10.1007/978-3-642-04441-0_70

[26] Schölkopf B, Tsuda K, Vert JP (2004) Kernel Methods in Computational Biology. MIT Press series on Computational Molecular Biology. 416 p.

[27] Wu KP, Wang SD (2009) Choosing the kernel parameters for support vector machines by the inter-cluster distance in the feature space. Pattern Recognit 42 (5) 710–717 10.1016/j.patcog.2008.08.030

[28] Frohlich H, Zell A (2005) Efficient parameter selection for support vector machines in classification and regression via model-based global optimization. Neural Networks (IJCNN). The 2005 International Joint Conference v3: 1431–1436 10.1109/IJCNN.2005.1556085

[29] He J, Hu HJ, Harrison R, Tai PC, Pan Y (2006) Rule Generation for Protein Secondary Structure Prediction with Support Vector Machines and Decision Tree. IEEE Trans Nanobioscience 5 (1) 46–53. 16570873 10.1109/tnb.2005.864021

[30] Dayem Ullah AZ, Lemoine NR, Chelala C (2012) SNPnexus: a web server for functional annotation of novel and publicly known genetic variants. Nucleic Acids Res 40 (Web Server issue) W65–70 10.1093/nar/gks364 22544707 PMC3394262

[31] Boyle AP, Hong EL, Hariharan M, Cheng Y, Schaub MA, et al (2012) Annotation of functional variation in personal genomes using RegulomeDB. Genome Res 22 (9) 1790–7 10.1101/gr.137323.112 22955989 PMC3431494

[32] Kleinmann N, Zaorsky NG, Showalter TN, Gomella LG, Lallas CD, et al (2012) The effect of ethnicity and sexual preference on prostate cancer related quality of life. Nat Rev Urol 9 (5) 258–65 10.1038/nrurol.2012.56 22487871

[33] Hoffman RM, Gilliland FD, Eley JW, Harlan LC, Stephenson RA, et al (2001) Racial and Ethnic Differences in Advanced-Stage Prostate Cancer: the Prostate Cancer Outcomes Study. JNCI J Natl Cancer Inst 93 (5) 388–395 10.1093/jnci/93.5.388 11238701

[34] Key TJ, Appleby PN, Reeves GK, Roddam A, Dorgan JF, et al (2003) Body mass index, serum sex hormones, and breast cancer risk in postmenopausal women. J Natl Cancer Inst 95 (16) 1218–26. 12928347 10.1093/jnci/djg022

[35] Chow WH, Blot WJ, Vaughan TL, Risch HA, Gammon MD, et al (1998) Body Mass Index and Risk of Adenocarcinomas of the Esophagus and Gastric Cardia. J Natl Cancer Inst 90 (2) 150–5. 9450576 10.1093/jnci/90.2.150

[36] Giovannucci E, Rimm EB, Liu Y, Leitzmann M, Wu K, et al (2003) Body Mass Index and Risk of Prostate Cancer in U.S. Health Professionals. J Natl Cancer Inst 95 (16) 1240–4. 12928350 10.1093/jnci/djg009

[37] Hiatt RA, Armstrong MA, Klatsky AL, Sidney S (1994) Alcohol consumption, smoking, and other risk factors and prostate cancer in a large health plan cohort in California (United States). Cancer Causes Control 5 (1) 66–72. 7510134 10.1007/BF01830728

[38] Caplan A, Kratz A (2002) Prostate-Specific Antigen and the Early Diagnosis of Prostate Cancer. Am J Clin Pathol (Suppl 1) S104–S108 10.1309/c4un-12lk-43hp-jxy3 14569806

[39] Aksoy Y, Oral A, Aksoy H, Demirel A, Akcay F (2003) PSA Density and PSA Transition Zone Density in the Diagnosis of Prostate Cancer in PSA Gray Zone Cases. Ann Clin Lab Sci 33 (3) 320–3. 12956448

[40] Hoffman RM, Gilliland FD, Adams-Cameron M, Hunt WC, Key CR (2002) Prostate-specific antigen testing accuracy in community practice. BMC Fam Pract 3: 19 10.1186/1471-2296-3-19 12398793 PMC137591

[41] Oesterling JE (1991) Prostate specific antigen: a critical assessment of the most useful tumor marker for adenocarcinoma of the prostate. J Urol 145 (5) 907–23. 1707989 10.1016/s0022-5347(17)38491-4

[42] Bretton PR, Evans WP, Borden JD, Castellanos RD (1994) The use of prostate specific antigen density to improve the sensitivity of prostate specific antigen in detecting prostate carcinoma. Cancer 74 (11) 2991–5. 7525040 10.1002/1097-0142(19941201)74:11<2991::aid-cncr2820741116>3.0.co;2-r

