# Correction: A Prostate Cancer Model Build by a Novel SVM-ID3 Hybrid Feature Selection Method Using Both Genotyping and Phenotype Data from dbGaP

**DOI:** 10.1371/journal.pone.0114956

**Published:** 2014-12-02

**Authors:** The PLOS ONE Staff

The following information is missing from the Acknowledgements section: Funding support for the GENEVA Prostate Cancer study was provided through the National Cancer Institute (R37CA54281, R01CA63464, P01CA33619, U01CA136792, U01CA98758, and RC2 CA148085) and the National Human Genome Research Institute (U01HG004726). Assistance with phenotype harmonization, SNP selection, data cleaning, meta-analyses, data management and dissemination, and general study coordination, was provided by the GENEVA Coordinating Center (U01HG004789-01).

## References

[1] Yücebaş SC, Aydın Son Y (2014) A Prostate Cancer Model Build by a Novel SVM-ID3 Hybrid Feature Selection Method Using Both Genotyping and Phenotype Data from dbGaP. PLoS ONE 9(3): e91404 doi:10.1371/journal.pone.0091404 24651484 10.1371/journal.pone.0091404 PMC3961262

