# Supplementary material for: A Prostate Cancer Model Build by a Novel SVM-ID3 Hybrid Feature Selection Method Using Both Genotyping and Phenotype Data from dbGaP
Source: PLoS One. 2014 Mar 20;9(3):e91404. doi: 10.1371/journal.pone.0091404 (PMC3961262; doi:10.1371/journal.pone.0091404)
Supplement: Table S1 — Whole list of SNPnexus results. (DOCX) [file pone.0091404.s001.docx]

## Table S1

| Gene | Entrez gene | Phenotype | Disease Class | Pubmed |
| --- | --- | --- | --- | --- |
| [GPR109A](http://geneticassociationdb.nih.gov/cgi-bin/tableview.cgi?table=diseaseview&cond=gene='GPR109A) | [338442](http://www.ncbi.nlm.nih.gov/sites/entrez?db=gene&cmd=Retrieve&dopt=full_report&list_uids=338442) | schizophrenia \| bipolar disorder | PSYCH | [19502010](http://www.ncbi.nlm.nih.gov/pubmed/19502010?) |
| [DDEF2](http://geneticassociationdb.nih.gov/cgi-bin/tableview.cgi?table=diseaseview&cond=gene='DDEF2) | [8853](http://www.ncbi.nlm.nih.gov/sites/entrez?db=gene&cmd=Retrieve&dopt=full_report&list_uids=8853) | multiple sclerosis | IMMUNE | [19010793](http://www.ncbi.nlm.nih.gov/pubmed/19010793?) |
| [SEMA5B](http://geneticassociationdb.nih.gov/cgi-bin/tableview.cgi?table=diseaseview&cond=gene='SEMA5B) | [54437](http://www.ncbi.nlm.nih.gov/sites/entrez?db=gene&cmd=Retrieve&dopt=full_report&list_uids=54437) | Type 2 Diabetes\| edema \| rosiglitazone | PHARMACOGENOMIC | [20628086](http://www.ncbi.nlm.nih.gov/pubmed/20628086?) |
| [SEMA5B](http://geneticassociationdb.nih.gov/cgi-bin/tableview.cgi?table=diseaseview&cond=gene='SEMA5B) | [54437](http://www.ncbi.nlm.nih.gov/sites/entrez?db=gene&cmd=Retrieve&dopt=full_report&list_uids=54437) | Tobacco Use Disorder | CHEMDEPENDENCY | [20379614](http://www.ncbi.nlm.nih.gov/pubmed/20379614?) |
| [MTAP](http://geneticassociationdb.nih.gov/cgi-bin/tableview.cgi?table=diseaseview&cond=gene='MTAP) | [4507](http://www.ncbi.nlm.nih.gov/sites/entrez?db=gene&cmd=Retrieve&dopt=full_report&list_uids=4507) | diabetes, type 2 | METABOLIC | [11985785](http://www.ncbi.nlm.nih.gov/pubmed/11985785?) |
| [MTAP](http://geneticassociationdb.nih.gov/cgi-bin/tableview.cgi?table=diseaseview&cond=gene='MTAP) | [4507](http://www.ncbi.nlm.nih.gov/sites/entrez?db=gene&cmd=Retrieve&dopt=full_report&list_uids=4507) | Melanoma\|Nevus\|Precancerous Conditions\|Skin Neoplasms | CANCER | [19578365](http://www.ncbi.nlm.nih.gov/pubmed/19578365?) |
| [MTAP](http://geneticassociationdb.nih.gov/cgi-bin/tableview.cgi?table=diseaseview&cond=gene='MTAP) | [4507](http://www.ncbi.nlm.nih.gov/sites/entrez?db=gene&cmd=Retrieve&dopt=full_report&list_uids=4507) | Brain Ischemia\|Diabetes Mellitus\|Hyperlipidemias\|Hypertension\|Intracranial Embolism | CARDIOVASCULAR | [19427650](http://www.ncbi.nlm.nih.gov/pubmed/19427650?) |
| [MTAP](http://geneticassociationdb.nih.gov/cgi-bin/tableview.cgi?table=diseaseview&cond=gene='MTAP) | [4507](http://www.ncbi.nlm.nih.gov/sites/entrez?db=gene&cmd=Retrieve&dopt=full_report&list_uids=4507) | Myocardial Infarction | CARDIOVASCULAR | [19272367](http://www.ncbi.nlm.nih.gov/pubmed/19272367?) |
| [MTAP](http://geneticassociationdb.nih.gov/cgi-bin/tableview.cgi?table=diseaseview&cond=gene='MTAP) | [4507](http://www.ncbi.nlm.nih.gov/sites/entrez?db=gene&cmd=Retrieve&dopt=full_report&list_uids=4507) |  | UNKNOWN | [19887491](http://www.ncbi.nlm.nih.gov/pubmed/19887491?) |
| [MTAP](http://geneticassociationdb.nih.gov/cgi-bin/tableview.cgi?table=diseaseview&cond=gene='MTAP) | [4507](http://www.ncbi.nlm.nih.gov/sites/entrez?db=gene&cmd=Retrieve&dopt=full_report&list_uids=4507) | Precursor Cell Lymphoblastic Leukemia-Lymphoma | CANCER | [19665068](http://www.ncbi.nlm.nih.gov/pubmed/19665068?) |
| [MTAP](http://geneticassociationdb.nih.gov/cgi-bin/tableview.cgi?table=diseaseview&cond=gene='MTAP) | [4507](http://www.ncbi.nlm.nih.gov/sites/entrez?db=gene&cmd=Retrieve&dopt=full_report&list_uids=4507) | Cutaneous nevi | OTHER | [19578365](http://www.ncbi.nlm.nih.gov/pubmed/19578365?) |
| [MTAP](http://geneticassociationdb.nih.gov/cgi-bin/tableview.cgi?table=diseaseview&cond=gene='MTAP) | [4507](http://www.ncbi.nlm.nih.gov/sites/entrez?db=gene&cmd=Retrieve&dopt=full_report&list_uids=4507) | melanoma\|Nevus\|Skin Neoplasms\|Sunburn | CANCER | [20647408](http://www.ncbi.nlm.nih.gov/pubmed/20647408?) |
| [MTAP](http://geneticassociationdb.nih.gov/cgi-bin/tableview.cgi?table=diseaseview&cond=gene='MTAP) | [4507](http://www.ncbi.nlm.nih.gov/sites/entrez?db=gene&cmd=Retrieve&dopt=full_report&list_uids=4507) | melanoma\|Nevus\|Skin Neoplasms | CANCER | [20574843](http://www.ncbi.nlm.nih.gov/pubmed/20574843?) |
| [CSMD1](http://geneticassociationdb.nih.gov/cgi-bin/tableview.cgi?table=diseaseview&cond=gene='CSMD1) | [64478](http://www.ncbi.nlm.nih.gov/sites/entrez?db=gene&cmd=Retrieve&dopt=full_report&list_uids=64478) | Tobacco Use Disorder | CHEMDEPENDENCY | [18519826](http://www.ncbi.nlm.nih.gov/pubmed/18519826?) |
| [CSMD1](http://geneticassociationdb.nih.gov/cgi-bin/tableview.cgi?table=diseaseview&cond=gene='CSMD1) | [64478](http://www.ncbi.nlm.nih.gov/sites/entrez?db=gene&cmd=Retrieve&dopt=full_report&list_uids=64478) | Chromosomal Instability\|Cystadenocarcinoma, Serous\|Ovarian Neoplasms | CANCER | [19383911](http://www.ncbi.nlm.nih.gov/pubmed/19383911?) |
| [CSMD1](http://geneticassociationdb.nih.gov/cgi-bin/tableview.cgi?table=diseaseview&cond=gene='CSMD1) | [64478](http://www.ncbi.nlm.nih.gov/sites/entrez?db=gene&cmd=Retrieve&dopt=full_report&list_uids=64478) | Celiac Disease\| | IMMUNE | [19240061](http://www.ncbi.nlm.nih.gov/pubmed/19240061?) |
| [CSMD1](http://geneticassociationdb.nih.gov/cgi-bin/tableview.cgi?table=diseaseview&cond=gene='CSMD1) | [64478](http://www.ncbi.nlm.nih.gov/sites/entrez?db=gene&cmd=Retrieve&dopt=full_report&list_uids=64478) | Mucocutaneous Lymph Node Syndrome | IMMUNE | [19132087](http://www.ncbi.nlm.nih.gov/pubmed/19132087?) |
| [CSMD1](http://geneticassociationdb.nih.gov/cgi-bin/tableview.cgi?table=diseaseview&cond=gene='CSMD1) | [64478](http://www.ncbi.nlm.nih.gov/sites/entrez?db=gene&cmd=Retrieve&dopt=full_report&list_uids=64478) | hypertension | CARDIOVASCULAR | [19960030](http://www.ncbi.nlm.nih.gov/pubmed/19960030?) |
| [CSMD1](http://geneticassociationdb.nih.gov/cgi-bin/tableview.cgi?table=diseaseview&cond=gene='CSMD1) | [64478](http://www.ncbi.nlm.nih.gov/sites/entrez?db=gene&cmd=Retrieve&dopt=full_report&list_uids=64478) | multiple sclerosis | IMMUNE | [19010793](http://www.ncbi.nlm.nih.gov/pubmed/19010793?) |
| [CSMD1](http://geneticassociationdb.nih.gov/cgi-bin/tableview.cgi?table=diseaseview&cond=gene='CSMD1) | [64478](http://www.ncbi.nlm.nih.gov/sites/entrez?db=gene&cmd=Retrieve&dopt=full_report&list_uids=64478) | smoking cessation | CHEMDEPENDENCY | [20235792](http://www.ncbi.nlm.nih.gov/pubmed/20235792?) |
| [CSMD1](http://geneticassociationdb.nih.gov/cgi-bin/tableview.cgi?table=diseaseview&cond=gene='CSMD1) | [64478](http://www.ncbi.nlm.nih.gov/sites/entrez?db=gene&cmd=Retrieve&dopt=full_report&list_uids=64478) | smoking cessation | CHEMDEPENDENCY | [20235792](http://www.ncbi.nlm.nih.gov/pubmed/20235792?) |
| [CSMD1](http://geneticassociationdb.nih.gov/cgi-bin/tableview.cgi?table=diseaseview&cond=gene='CSMD1) | [64478](http://www.ncbi.nlm.nih.gov/sites/entrez?db=gene&cmd=Retrieve&dopt=full_report&list_uids=64478) | Psoriasis | IMMUNE | [20953187](http://www.ncbi.nlm.nih.gov/pubmed/20953187?) |
| [CSMD1](http://geneticassociationdb.nih.gov/cgi-bin/tableview.cgi?table=diseaseview&cond=gene='CSMD1) | [64478](http://www.ncbi.nlm.nih.gov/sites/entrez?db=gene&cmd=Retrieve&dopt=full_report&list_uids=64478) | Peripheral Vascular Diseases | CARDIOVASCULAR | [20610895](http://www.ncbi.nlm.nih.gov/pubmed/20610895?) |
| [CSMD1](http://geneticassociationdb.nih.gov/cgi-bin/tableview.cgi?table=diseaseview&cond=gene='CSMD1) | [64478](http://www.ncbi.nlm.nih.gov/sites/entrez?db=gene&cmd=Retrieve&dopt=full_report&list_uids=64478) | Tobacco Use Disorder | CHEMDEPENDENCY | [20379614](http://www.ncbi.nlm.nih.gov/pubmed/20379614?) |
| [TNFAIP1](http://geneticassociationdb.nih.gov/cgi-bin/tableview.cgi?table=diseaseview&cond=gene='TNFAIP1) | [7126](http://www.ncbi.nlm.nih.gov/sites/entrez?db=gene&cmd=Retrieve&dopt=full_report&list_uids=7126) | anorexia nervosa | PSYCH | [11702059](http://www.ncbi.nlm.nih.gov/pubmed/11702059?) |
| [TNFAIP1](http://geneticassociationdb.nih.gov/cgi-bin/tableview.cgi?table=diseaseview&cond=gene='TNFAIP1) | [7126](http://www.ncbi.nlm.nih.gov/sites/entrez?db=gene&cmd=Retrieve&dopt=full_report&list_uids=7126) | Malaria infection | INFECTION | [11929592](http://www.ncbi.nlm.nih.gov/pubmed/11929592?) |
| [TNFAIP1](http://geneticassociationdb.nih.gov/cgi-bin/tableview.cgi?table=diseaseview&cond=gene='TNFAIP1) | [7126](http://www.ncbi.nlm.nih.gov/sites/entrez?db=gene&cmd=Retrieve&dopt=full_report&list_uids=7126) | arthritis, rheumatoid | IMMUNE | [11791643](http://www.ncbi.nlm.nih.gov/pubmed/11791643?) |
| [TNFAIP1](http://geneticassociationdb.nih.gov/cgi-bin/tableview.cgi?table=diseaseview&cond=gene='TNFAIP1) | [7126](http://www.ncbi.nlm.nih.gov/sites/entrez?db=gene&cmd=Retrieve&dopt=full_report&list_uids=7126) | systemic lupus erythematosus | IMMUNE | [11704801](http://www.ncbi.nlm.nih.gov/pubmed/11704801?) |
| [TNFAIP1](http://geneticassociationdb.nih.gov/cgi-bin/tableview.cgi?table=diseaseview&cond=gene='TNFAIP1) | [7126](http://www.ncbi.nlm.nih.gov/sites/entrez?db=gene&cmd=Retrieve&dopt=full_report&list_uids=7126) | nephropathy, IgA | RENAL | [11849463](http://www.ncbi.nlm.nih.gov/pubmed/11849463?) |
| [TNFAIP1](http://geneticassociationdb.nih.gov/cgi-bin/tableview.cgi?table=diseaseview&cond=gene='TNFAIP1) | [7126](http://www.ncbi.nlm.nih.gov/sites/entrez?db=gene&cmd=Retrieve&dopt=full_report&list_uids=7126) | sarcoidosis | IMMUNE | [12039524](http://www.ncbi.nlm.nih.gov/pubmed/12039524?) |
| [TNFAIP1](http://geneticassociationdb.nih.gov/cgi-bin/tableview.cgi?table=diseaseview&cond=gene='TNFAIP1) | [7126](http://www.ncbi.nlm.nih.gov/sites/entrez?db=gene&cmd=Retrieve&dopt=full_report&list_uids=7126) | Asthma\|Obesity | METABOLIC | [19196817](http://www.ncbi.nlm.nih.gov/pubmed/19196817?) |
| [TNFAIP1](http://geneticassociationdb.nih.gov/cgi-bin/tableview.cgi?table=diseaseview&cond=gene='TNFAIP1) | [7126](http://www.ncbi.nlm.nih.gov/sites/entrez?db=gene&cmd=Retrieve&dopt=full_report&list_uids=7126) | Alzheimer's disease | NEUROLOGICAL | [19141999](http://www.ncbi.nlm.nih.gov/pubmed/19141999?) |
| [ATP2B2](http://geneticassociationdb.nih.gov/cgi-bin/tableview.cgi?table=diseaseview&cond=gene='ATP2B2) | [491](http://www.ncbi.nlm.nih.gov/sites/entrez?db=gene&cmd=Retrieve&dopt=full_report&list_uids=491) | schizophrenia | PSYCH | [19850283](http://www.ncbi.nlm.nih.gov/pubmed/19850283?) |
| [ATP2B2](http://geneticassociationdb.nih.gov/cgi-bin/tableview.cgi?table=diseaseview&cond=gene='ATP2B2) | [491](http://www.ncbi.nlm.nih.gov/sites/entrez?db=gene&cmd=Retrieve&dopt=full_report&list_uids=491) | serum metabolites | METABOLIC | [19043545](http://www.ncbi.nlm.nih.gov/pubmed/19043545?) |
| [ATP2B2](http://geneticassociationdb.nih.gov/cgi-bin/tableview.cgi?table=diseaseview&cond=gene='ATP2B2) | [491](http://www.ncbi.nlm.nih.gov/sites/entrez?db=gene&cmd=Retrieve&dopt=full_report&list_uids=491) | Type 2 Diabetes\| edema \| rosiglitazone | PHARMACOGENOMIC | [20628086](http://www.ncbi.nlm.nih.gov/pubmed/20628086?) |
| [ATP2B2](http://geneticassociationdb.nih.gov/cgi-bin/tableview.cgi?table=diseaseview&cond=gene='ATP2B2) | [491](http://www.ncbi.nlm.nih.gov/sites/entrez?db=gene&cmd=Retrieve&dopt=full_report&list_uids=491) | Tobacco Use Disorder | CHEMDEPENDENCY | [20379614](http://www.ncbi.nlm.nih.gov/pubmed/20379614?) |
| [FCAMR](http://geneticassociationdb.nih.gov/cgi-bin/tableview.cgi?table=diseaseview&cond=gene='FCAMR) | [83953](http://www.ncbi.nlm.nih.gov/sites/entrez?db=gene&cmd=Retrieve&dopt=full_report&list_uids=83953) | Tobacco Use Disorder | CHEMDEPENDENCY | [20379614](http://www.ncbi.nlm.nih.gov/pubmed/20379614?) |
| [DPT](http://geneticassociationdb.nih.gov/cgi-bin/tableview.cgi?table=diseaseview&cond=gene='DPT) | [1805](http://www.ncbi.nlm.nih.gov/sites/entrez?db=gene&cmd=Retrieve&dopt=full_report&list_uids=1805) | Cardiovascular Diseases | CARDIOVASCULAR | [17903295](http://www.ncbi.nlm.nih.gov/pubmed/17903295?) |
| [DPT](http://geneticassociationdb.nih.gov/cgi-bin/tableview.cgi?table=diseaseview&cond=gene='DPT) | [1805](http://www.ncbi.nlm.nih.gov/sites/entrez?db=gene&cmd=Retrieve&dopt=full_report&list_uids=1805) | Hypertension | CARDIOVASCULAR | [19536175](http://www.ncbi.nlm.nih.gov/pubmed/19536175?) |
| [DPT](http://geneticassociationdb.nih.gov/cgi-bin/tableview.cgi?table=diseaseview&cond=gene='DPT) | [1805](http://www.ncbi.nlm.nih.gov/sites/entrez?db=gene&cmd=Retrieve&dopt=full_report&list_uids=1805) | Osteoporosis | METABOLIC | [19064610](http://www.ncbi.nlm.nih.gov/pubmed/19064610?) |
| [DPT](http://geneticassociationdb.nih.gov/cgi-bin/tableview.cgi?table=diseaseview&cond=gene='DPT) | [1805](http://www.ncbi.nlm.nih.gov/sites/entrez?db=gene&cmd=Retrieve&dopt=full_report&list_uids=1805) | morbidity-free survival | AGING | [17903295](http://www.ncbi.nlm.nih.gov/pubmed/17903295?) |
| [USP24](http://geneticassociationdb.nih.gov/cgi-bin/tableview.cgi?table=diseaseview&cond=gene='USP24) | [23358](http://www.ncbi.nlm.nih.gov/sites/entrez?db=gene&cmd=Retrieve&dopt=full_report&list_uids=23358) | Parkinson's disease | NEUROLOGICAL | [16917932](http://www.ncbi.nlm.nih.gov/pubmed/16917932?) |
| [USP24](http://geneticassociationdb.nih.gov/cgi-bin/tableview.cgi?table=diseaseview&cond=gene='USP24) | [23358](http://www.ncbi.nlm.nih.gov/sites/entrez?db=gene&cmd=Retrieve&dopt=full_report&list_uids=23358) | Parkinson's disease | NEUROLOGICAL | [20302855](http://www.ncbi.nlm.nih.gov/pubmed/20302855?) |
| [NCAM2](http://geneticassociationdb.nih.gov/cgi-bin/tableview.cgi?table=diseaseview&cond=gene='NCAM2) | [4685](http://www.ncbi.nlm.nih.gov/sites/entrez?db=gene&cmd=Retrieve&dopt=full_report&list_uids=4685) | several psychiatric disorders | PSYCH | [19086053](http://www.ncbi.nlm.nih.gov/pubmed/19086053?) |
| [NCAM2](http://geneticassociationdb.nih.gov/cgi-bin/tableview.cgi?table=diseaseview&cond=gene='NCAM2) | [4685](http://www.ncbi.nlm.nih.gov/sites/entrez?db=gene&cmd=Retrieve&dopt=full_report&list_uids=4685) | Alzheimer Disease\|Alzheimer's Disease | NEUROLOGICAL | [20932310](http://www.ncbi.nlm.nih.gov/pubmed/20932310?) |
| [NCAM2](http://geneticassociationdb.nih.gov/cgi-bin/tableview.cgi?table=diseaseview&cond=gene='NCAM2) | [4685](http://www.ncbi.nlm.nih.gov/sites/entrez?db=gene&cmd=Retrieve&dopt=full_report&list_uids=4685) | Tobacco Use Disorder | CHEMDEPENDENCY | [20379614](http://www.ncbi.nlm.nih.gov/pubmed/20379614?) |
| [ST6GALNAC3](http://geneticassociationdb.nih.gov/cgi-bin/tableview.cgi?table=diseaseview&cond=gene='ST6GALNAC3) | [256435](http://www.ncbi.nlm.nih.gov/sites/entrez?db=gene&cmd=Retrieve&dopt=full_report&list_uids=256435) | Tobacco Use Disorder | CHEMDEPENDENCY | [18519826](http://www.ncbi.nlm.nih.gov/pubmed/18519826?) |
| [ST6GALNAC3](http://geneticassociationdb.nih.gov/cgi-bin/tableview.cgi?table=diseaseview&cond=gene='ST6GALNAC3) | [256435](http://www.ncbi.nlm.nih.gov/sites/entrez?db=gene&cmd=Retrieve&dopt=full_report&list_uids=256435) | Tobacco Use Disorder | CHEMDEPENDENCY | [20379614](http://www.ncbi.nlm.nih.gov/pubmed/20379614?) |
| [ST6GALNAC3](http://geneticassociationdb.nih.gov/cgi-bin/tableview.cgi?table=diseaseview&cond=gene='ST6GALNAC3) | [256435](http://www.ncbi.nlm.nih.gov/sites/entrez?db=gene&cmd=Retrieve&dopt=full_report&list_uids=256435) | Alcoholism | CHEMDEPENDENCY | [20421487](http://www.ncbi.nlm.nih.gov/pubmed/20421487?) |
| [IL1RAPL1](http://geneticassociationdb.nih.gov/cgi-bin/tableview.cgi?table=diseaseview&cond=gene='IL1RAPL1) | [11141](http://www.ncbi.nlm.nih.gov/sites/entrez?db=gene&cmd=Retrieve&dopt=full_report&list_uids=11141) | schizophrenia \| autism | DEVELOPMENTAL | [19736351](http://www.ncbi.nlm.nih.gov/pubmed/19736351?) |
| [IL1RAPL1](http://geneticassociationdb.nih.gov/cgi-bin/tableview.cgi?table=diseaseview&cond=gene='IL1RAPL1) | [11141](http://www.ncbi.nlm.nih.gov/sites/entrez?db=gene&cmd=Retrieve&dopt=full_report&list_uids=11141) | cognitive ability | NEUROLOGICAL | [18467032](http://www.ncbi.nlm.nih.gov/pubmed/18467032?) |
| [IL1RAPL1](http://geneticassociationdb.nih.gov/cgi-bin/tableview.cgi?table=diseaseview&cond=gene='IL1RAPL1) | [11141](http://www.ncbi.nlm.nih.gov/sites/entrez?db=gene&cmd=Retrieve&dopt=full_report&list_uids=11141) | Chorioamnionitis\|Fetal Membranes, Premature Rupture\|Infection of amniotic sac and membranes\|Obstetric Labor, Premature\|Pre-Eclampsia\|Premature Birth | REPRODUCTION | [20452482](http://www.ncbi.nlm.nih.gov/pubmed/20452482?) |
| [IL1RAPL1](http://geneticassociationdb.nih.gov/cgi-bin/tableview.cgi?table=diseaseview&cond=gene='IL1RAPL1) | [11141](http://www.ncbi.nlm.nih.gov/sites/entrez?db=gene&cmd=Retrieve&dopt=full_report&list_uids=11141) | Chorioamnionitis\|Fetal Membranes, Premature Rupture\|Infection of amniotic sac and membranes | REPRODUCTION | [20673868](http://www.ncbi.nlm.nih.gov/pubmed/20673868?) |
| [IL1RAPL1](http://geneticassociationdb.nih.gov/cgi-bin/tableview.cgi?table=diseaseview&cond=gene='IL1RAPL1) | [11141](http://www.ncbi.nlm.nih.gov/sites/entrez?db=gene&cmd=Retrieve&dopt=full_report&list_uids=11141) | Type 2 Diabetes\| edema \| rosiglitazone | PHARMACOGENOMIC | [20628086](http://www.ncbi.nlm.nih.gov/pubmed/20628086?) |
| [ECOP](http://geneticassociationdb.nih.gov/cgi-bin/tableview.cgi?table=diseaseview&cond=gene='ECOP) |  | Apoplexy\|Cerebral Hemorrhage\|Cerebral Hemorrhages\|Intracranial Hemorrhages\|Stroke\|Subarachnoid Hemorrhage | CARDIOVASCULAR | [20198315](http://www.ncbi.nlm.nih.gov/pubmed/20198315?) |
| [SHB](http://geneticassociationdb.nih.gov/cgi-bin/tableview.cgi?table=diseaseview&cond=gene='SHB) | [6461](http://www.ncbi.nlm.nih.gov/sites/entrez?db=gene&cmd=Retrieve&dopt=full_report&list_uids=6461) | Alzheimer's disease | NEUROLOGICAL | [19141999](http://www.ncbi.nlm.nih.gov/pubmed/19141999?) |
| [SHB](http://geneticassociationdb.nih.gov/cgi-bin/tableview.cgi?table=diseaseview&cond=gene='SHB) | [6461](http://www.ncbi.nlm.nih.gov/sites/entrez?db=gene&cmd=Retrieve&dopt=full_report&list_uids=6461) | Brain structure | NEUROLOGICAL | [20171287](http://www.ncbi.nlm.nih.gov/pubmed/20171287?) |
| [IMMP2L](http://geneticassociationdb.nih.gov/cgi-bin/tableview.cgi?table=diseaseview&cond=gene='IMMP2L) | [83943](http://www.ncbi.nlm.nih.gov/sites/entrez?db=gene&cmd=Retrieve&dopt=full_report&list_uids=83943) | Autism | PSYCH | [19401682](http://www.ncbi.nlm.nih.gov/pubmed/19401682?) |
| [IMMP2L](http://geneticassociationdb.nih.gov/cgi-bin/tableview.cgi?table=diseaseview&cond=gene='IMMP2L) | [83943](http://www.ncbi.nlm.nih.gov/sites/entrez?db=gene&cmd=Retrieve&dopt=full_report&list_uids=83943) | Celiac Disease\| | IMMUNE | [19240061](http://www.ncbi.nlm.nih.gov/pubmed/19240061?) |
| [IMMP2L](http://geneticassociationdb.nih.gov/cgi-bin/tableview.cgi?table=diseaseview&cond=gene='IMMP2L) | [83943](http://www.ncbi.nlm.nih.gov/sites/entrez?db=gene&cmd=Retrieve&dopt=full_report&list_uids=83943) | Autism | PSYCH | [19058789](http://www.ncbi.nlm.nih.gov/pubmed/19058789?) |
| [IMMP2L](http://geneticassociationdb.nih.gov/cgi-bin/tableview.cgi?table=diseaseview&cond=gene='IMMP2L) | [83943](http://www.ncbi.nlm.nih.gov/sites/entrez?db=gene&cmd=Retrieve&dopt=full_report&list_uids=83943) | ADHD \| attention-deficit hyperactivity disorder | PSYCH | [19546859](http://www.ncbi.nlm.nih.gov/pubmed/19546859?) |
| [IMMP2L](http://geneticassociationdb.nih.gov/cgi-bin/tableview.cgi?table=diseaseview&cond=gene='IMMP2L) | [83943](http://www.ncbi.nlm.nih.gov/sites/entrez?db=gene&cmd=Retrieve&dopt=full_report&list_uids=83943) | Cognitive performance | NEUROLOGICAL | [19734545](http://www.ncbi.nlm.nih.gov/pubmed/19734545?) |
| [IMMP2L](http://geneticassociationdb.nih.gov/cgi-bin/tableview.cgi?table=diseaseview&cond=gene='IMMP2L) | [83943](http://www.ncbi.nlm.nih.gov/sites/entrez?db=gene&cmd=Retrieve&dopt=full_report&list_uids=83943) | Cognitive performance | NEUROLOGICAL | [19734545](http://www.ncbi.nlm.nih.gov/pubmed/19734545?) |
| [IMMP2L](http://geneticassociationdb.nih.gov/cgi-bin/tableview.cgi?table=diseaseview&cond=gene='IMMP2L) | [83943](http://www.ncbi.nlm.nih.gov/sites/entrez?db=gene&cmd=Retrieve&dopt=full_report&list_uids=83943) | Acquired Immunodeficiency Syndrome\|Disease Progression | INFECTION | [20877624](http://www.ncbi.nlm.nih.gov/pubmed/20877624?) |
| [IMMP2L](http://geneticassociationdb.nih.gov/cgi-bin/tableview.cgi?table=diseaseview&cond=gene='IMMP2L) | [83943](http://www.ncbi.nlm.nih.gov/sites/entrez?db=gene&cmd=Retrieve&dopt=full_report&list_uids=83943) | Psychiatric Disorders | PSYCH | [20398908](http://www.ncbi.nlm.nih.gov/pubmed/20398908?) |
| [IMMP2L](http://geneticassociationdb.nih.gov/cgi-bin/tableview.cgi?table=diseaseview&cond=gene='IMMP2L) | [83943](http://www.ncbi.nlm.nih.gov/sites/entrez?db=gene&cmd=Retrieve&dopt=full_report&list_uids=83943) | Tobacco Use Disorder | CHEMDEPENDENCY | [20379614](http://www.ncbi.nlm.nih.gov/pubmed/20379614?) |
| [COBL](http://geneticassociationdb.nih.gov/cgi-bin/tableview.cgi?table=diseaseview&cond=gene='COBL) | [23242](http://www.ncbi.nlm.nih.gov/sites/entrez?db=gene&cmd=Retrieve&dopt=full_report&list_uids=23242) | Celiac Disease\| | IMMUNE | [19240061](http://www.ncbi.nlm.nih.gov/pubmed/19240061?) |
| [COBL](http://geneticassociationdb.nih.gov/cgi-bin/tableview.cgi?table=diseaseview&cond=gene='COBL) | [23242](http://www.ncbi.nlm.nih.gov/sites/entrez?db=gene&cmd=Retrieve&dopt=full_report&list_uids=23242) | Autism | PSYCH | [19058789](http://www.ncbi.nlm.nih.gov/pubmed/19058789?) |
| [COBL](http://geneticassociationdb.nih.gov/cgi-bin/tableview.cgi?table=diseaseview&cond=gene='COBL) | [23242](http://www.ncbi.nlm.nih.gov/sites/entrez?db=gene&cmd=Retrieve&dopt=full_report&list_uids=23242) | Tobacco Use Disorder | CHEMDEPENDENCY | [20379614](http://www.ncbi.nlm.nih.gov/pubmed/20379614?) |
| [SPTBN1](http://geneticassociationdb.nih.gov/cgi-bin/tableview.cgi?table=diseaseview&cond=gene='SPTBN1) | [6711](http://www.ncbi.nlm.nih.gov/sites/entrez?db=gene&cmd=Retrieve&dopt=full_report&list_uids=6711) | Fractures, Bone | METABOLIC | [19801982](http://www.ncbi.nlm.nih.gov/pubmed/19801982?) |
| [SPTBN1](http://geneticassociationdb.nih.gov/cgi-bin/tableview.cgi?table=diseaseview&cond=gene='SPTBN1) | [6711](http://www.ncbi.nlm.nih.gov/sites/entrez?db=gene&cmd=Retrieve&dopt=full_report&list_uids=6711) | Dengue Hemorrhagic Fever | INFECTION | [20588308](http://www.ncbi.nlm.nih.gov/pubmed/20588308?) |
| [SPTBN1](http://geneticassociationdb.nih.gov/cgi-bin/tableview.cgi?table=diseaseview&cond=gene='SPTBN1) | [6711](http://www.ncbi.nlm.nih.gov/sites/entrez?db=gene&cmd=Retrieve&dopt=full_report&list_uids=6711) | Osteoporosis | METABOLIC | [20554715](http://www.ncbi.nlm.nih.gov/pubmed/20554715?) |
| [SPTBN1](http://geneticassociationdb.nih.gov/cgi-bin/tableview.cgi?table=diseaseview&cond=gene='SPTBN1) | [6711](http://www.ncbi.nlm.nih.gov/sites/entrez?db=gene&cmd=Retrieve&dopt=full_report&list_uids=6711) | Tobacco Use Disorder | CHEMDEPENDENCY | [20379614](http://www.ncbi.nlm.nih.gov/pubmed/20379614?) |
| [SPTBN1](http://geneticassociationdb.nih.gov/cgi-bin/tableview.cgi?table=diseaseview&cond=gene='SPTBN1) | [6711](http://www.ncbi.nlm.nih.gov/sites/entrez?db=gene&cmd=Retrieve&dopt=full_report&list_uids=6711) | HIV Infections\|[X]Human immunodeficiency virus disease | INFECTION | [21083371](http://www.ncbi.nlm.nih.gov/pubmed/21083371?) |
| [LRRTM4](http://geneticassociationdb.nih.gov/cgi-bin/tableview.cgi?table=diseaseview&cond=gene='LRRTM4) | [80059](http://www.ncbi.nlm.nih.gov/sites/entrez?db=gene&cmd=Retrieve&dopt=full_report&list_uids=80059) | Tobacco Use Disorder | CHEMDEPENDENCY | [20379614](http://www.ncbi.nlm.nih.gov/pubmed/20379614?) |
| [GPR109A](http://geneticassociationdb.nih.gov/cgi-bin/tableview.cgi?table=diseaseview&cond=gene='GPR109A) | [338442](http://www.ncbi.nlm.nih.gov/sites/entrez?db=gene&cmd=Retrieve&dopt=full_report&list_uids=338442) | schizophrenia \| bipolar disorder | PSYCH | [19502010](http://www.ncbi.nlm.nih.gov/pubmed/19502010?) |
| [CPM](http://geneticassociationdb.nih.gov/cgi-bin/tableview.cgi?table=diseaseview&cond=gene='CPM) | [1368](http://www.ncbi.nlm.nih.gov/sites/entrez?db=gene&cmd=Retrieve&dopt=full_report&list_uids=1368) | bronchodilator response | IMMUNE | [18617639](http://www.ncbi.nlm.nih.gov/pubmed/18617639?) |
| [SMARCA4](http://geneticassociationdb.nih.gov/cgi-bin/tableview.cgi?table=diseaseview&cond=gene='SMARCA4) | [6597](http://www.ncbi.nlm.nih.gov/sites/entrez?db=gene&cmd=Retrieve&dopt=full_report&list_uids=6597) | Cardiovascular Diseases | CARDIOVASCULAR | [19913121](http://www.ncbi.nlm.nih.gov/pubmed/19913121?) |
| [SMARCA4](http://geneticassociationdb.nih.gov/cgi-bin/tableview.cgi?table=diseaseview&cond=gene='SMARCA4) | [6597](http://www.ncbi.nlm.nih.gov/sites/entrez?db=gene&cmd=Retrieve&dopt=full_report&list_uids=6597) | breast cancer | CANCER | [19183483](http://www.ncbi.nlm.nih.gov/pubmed/19183483?) |
| [SMARCA4](http://geneticassociationdb.nih.gov/cgi-bin/tableview.cgi?table=diseaseview&cond=gene='SMARCA4) | [6597](http://www.ncbi.nlm.nih.gov/sites/entrez?db=gene&cmd=Retrieve&dopt=full_report&list_uids=6597) | plasma HDL cholesterol (HDL-C) levels | METABOLIC | [18660489](http://www.ncbi.nlm.nih.gov/pubmed/18660489?) |
| [SMARCA4](http://geneticassociationdb.nih.gov/cgi-bin/tableview.cgi?table=diseaseview&cond=gene='SMARCA4) | [6597](http://www.ncbi.nlm.nih.gov/sites/entrez?db=gene&cmd=Retrieve&dopt=full_report&list_uids=6597) | Type 2 Diabetes\| edema \| rosiglitazone | PHARMACOGENOMIC | [20628086](http://www.ncbi.nlm.nih.gov/pubmed/20628086?) |
| [SMARCA4](http://geneticassociationdb.nih.gov/cgi-bin/tableview.cgi?table=diseaseview&cond=gene='SMARCA4) | [6597](http://www.ncbi.nlm.nih.gov/sites/entrez?db=gene&cmd=Retrieve&dopt=full_report&list_uids=6597) | Coronary Artery Disease | CARDIOVASCULAR | [20810930](http://www.ncbi.nlm.nih.gov/pubmed/20810930?) |
| [ANGPT2](http://geneticassociationdb.nih.gov/cgi-bin/tableview.cgi?table=diseaseview&cond=gene='ANGPT2) | [285](http://www.ncbi.nlm.nih.gov/sites/entrez?db=gene&cmd=Retrieve&dopt=full_report&list_uids=285) | pregnancy loss, recurrent | OTHER | [14556828](http://www.ncbi.nlm.nih.gov/pubmed/14556828?) |
| [ANGPT2](http://geneticassociationdb.nih.gov/cgi-bin/tableview.cgi?table=diseaseview&cond=gene='ANGPT2) | [285](http://www.ncbi.nlm.nih.gov/sites/entrez?db=gene&cmd=Retrieve&dopt=full_report&list_uids=285) | fetal loss, late | REPRODUCTION | [15694966](http://www.ncbi.nlm.nih.gov/pubmed/15694966?) |
| [ANGPT2](http://geneticassociationdb.nih.gov/cgi-bin/tableview.cgi?table=diseaseview&cond=gene='ANGPT2) | [285](http://www.ncbi.nlm.nih.gov/sites/entrez?db=gene&cmd=Retrieve&dopt=full_report&list_uids=285) | uterine leiomyomas | OTHER | [16009172](http://www.ncbi.nlm.nih.gov/pubmed/16009172?) |
| [ANGPT2](http://geneticassociationdb.nih.gov/cgi-bin/tableview.cgi?table=diseaseview&cond=gene='ANGPT2) | [285](http://www.ncbi.nlm.nih.gov/sites/entrez?db=gene&cmd=Retrieve&dopt=full_report&list_uids=285) | idiopathic recurrent miscarriage | OTHER | [14556828](http://www.ncbi.nlm.nih.gov/pubmed/14556828?) |
| [ANGPT2](http://geneticassociationdb.nih.gov/cgi-bin/tableview.cgi?table=diseaseview&cond=gene='ANGPT2) | [285](http://www.ncbi.nlm.nih.gov/sites/entrez?db=gene&cmd=Retrieve&dopt=full_report&list_uids=285) | retinopathy of prematurity | VISION | [16877277](http://www.ncbi.nlm.nih.gov/pubmed/16877277?) |
| [MCPH1](http://geneticassociationdb.nih.gov/cgi-bin/tableview.cgi?table=diseaseview&cond=gene='MCPH1) | [79648](http://www.ncbi.nlm.nih.gov/sites/entrez?db=gene&cmd=Retrieve&dopt=full_report&list_uids=79648) | brain size | NEUROLOGICAL | [17566767](http://www.ncbi.nlm.nih.gov/pubmed/17566767?) |
| [MCPH1](http://geneticassociationdb.nih.gov/cgi-bin/tableview.cgi?table=diseaseview&cond=gene='MCPH1) | [79648](http://www.ncbi.nlm.nih.gov/sites/entrez?db=gene&cmd=Retrieve&dopt=full_report&list_uids=79648) | cognitive function head circumference social intelligence | PSYCH | [17251122](http://www.ncbi.nlm.nih.gov/pubmed/17251122?) |
| [MCPH1](http://geneticassociationdb.nih.gov/cgi-bin/tableview.cgi?table=diseaseview&cond=gene='MCPH1) | [79648](http://www.ncbi.nlm.nih.gov/sites/entrez?db=gene&cmd=Retrieve&dopt=full_report&list_uids=79648) | Mental Retardation\|Microcephaly | DEVELOPMENTAL | [19267414](http://www.ncbi.nlm.nih.gov/pubmed/19267414?) |
| [MCPH1](http://geneticassociationdb.nih.gov/cgi-bin/tableview.cgi?table=diseaseview&cond=gene='MCPH1) | [79648](http://www.ncbi.nlm.nih.gov/sites/entrez?db=gene&cmd=Retrieve&dopt=full_report&list_uids=79648) | Coronary Artery Disease\| | CARDIOVASCULAR | [18651322](http://www.ncbi.nlm.nih.gov/pubmed/18651322?) |
| [MCPH1](http://geneticassociationdb.nih.gov/cgi-bin/tableview.cgi?table=diseaseview&cond=gene='MCPH1) | [79648](http://www.ncbi.nlm.nih.gov/sites/entrez?db=gene&cmd=Retrieve&dopt=full_report&list_uids=79648) | Adenocarcinoma\|Pancreatic Neoplasms | CANCER | [19690177](http://www.ncbi.nlm.nih.gov/pubmed/19690177?) |
| [MCPH1](http://geneticassociationdb.nih.gov/cgi-bin/tableview.cgi?table=diseaseview&cond=gene='MCPH1) | [79648](http://www.ncbi.nlm.nih.gov/sites/entrez?db=gene&cmd=Retrieve&dopt=full_report&list_uids=79648) | Multiple System Atrophy | UNKNOWN | [19475667](http://www.ncbi.nlm.nih.gov/pubmed/19475667?) |
| [ANGPT2](http://geneticassociationdb.nih.gov/cgi-bin/tableview.cgi?table=diseaseview&cond=gene='ANGPT2) | [285](http://www.ncbi.nlm.nih.gov/sites/entrez?db=gene&cmd=Retrieve&dopt=full_report&list_uids=285) | Lymphedema | HEMATOLOGICAL | [18564921](http://www.ncbi.nlm.nih.gov/pubmed/18564921?) |
| [ANGPT2](http://geneticassociationdb.nih.gov/cgi-bin/tableview.cgi?table=diseaseview&cond=gene='ANGPT2) | [285](http://www.ncbi.nlm.nih.gov/sites/entrez?db=gene&cmd=Retrieve&dopt=full_report&list_uids=285) | Birth Weight\|Retinopathy of Prematurity | METABOLIC | [19018553](http://www.ncbi.nlm.nih.gov/pubmed/19018553?) |
| [ANGPT2](http://geneticassociationdb.nih.gov/cgi-bin/tableview.cgi?table=diseaseview&cond=gene='ANGPT2) | [285](http://www.ncbi.nlm.nih.gov/sites/entrez?db=gene&cmd=Retrieve&dopt=full_report&list_uids=285) | BMI- Edema rosiglitazone or pioglitazone | PHARMACOGENOMIC | [18996102](http://www.ncbi.nlm.nih.gov/pubmed/18996102?) |
| [ANGPT2](http://geneticassociationdb.nih.gov/cgi-bin/tableview.cgi?table=diseaseview&cond=gene='ANGPT2) | [285](http://www.ncbi.nlm.nih.gov/sites/entrez?db=gene&cmd=Retrieve&dopt=full_report&list_uids=285) | Retinopathy of Prematurity | VISION | [18568888](http://www.ncbi.nlm.nih.gov/pubmed/18568888?) |
| [ANGPT2](http://geneticassociationdb.nih.gov/cgi-bin/tableview.cgi?table=diseaseview&cond=gene='ANGPT2) | [285](http://www.ncbi.nlm.nih.gov/sites/entrez?db=gene&cmd=Retrieve&dopt=full_report&list_uids=285) | Stroke | CARDIOVASCULAR | [19341361](http://www.ncbi.nlm.nih.gov/pubmed/19341361?) |
| [ANGPT2](http://geneticassociationdb.nih.gov/cgi-bin/tableview.cgi?table=diseaseview&cond=gene='ANGPT2) | [285](http://www.ncbi.nlm.nih.gov/sites/entrez?db=gene&cmd=Retrieve&dopt=full_report&list_uids=285) | Respiratory Distress Syndrome, Adult | UNKNOWN | [19271210](http://www.ncbi.nlm.nih.gov/pubmed/19271210?) |
| [MCPH1](http://geneticassociationdb.nih.gov/cgi-bin/tableview.cgi?table=diseaseview&cond=gene='MCPH1) | [79648](http://www.ncbi.nlm.nih.gov/sites/entrez?db=gene&cmd=Retrieve&dopt=full_report&list_uids=79648) | atherosclerosis | CARDIOVASCULAR | [20485444](http://www.ncbi.nlm.nih.gov/pubmed/20485444?) |
| [MCPH1](http://geneticassociationdb.nih.gov/cgi-bin/tableview.cgi?table=diseaseview&cond=gene='MCPH1) | [79648](http://www.ncbi.nlm.nih.gov/sites/entrez?db=gene&cmd=Retrieve&dopt=full_report&list_uids=79648) | Tobacco Use Disorder | CHEMDEPENDENCY | [20379614](http://www.ncbi.nlm.nih.gov/pubmed/20379614?) |
| [MCPH1](http://geneticassociationdb.nih.gov/cgi-bin/tableview.cgi?table=diseaseview&cond=gene='MCPH1) | [79648](http://www.ncbi.nlm.nih.gov/sites/entrez?db=gene&cmd=Retrieve&dopt=full_report&list_uids=79648) | Micrencephaly \|Microcephaly | DEVELOPMENTAL | [20080800](http://www.ncbi.nlm.nih.gov/pubmed/20080800?) |
| [MCPH1](http://geneticassociationdb.nih.gov/cgi-bin/tableview.cgi?table=diseaseview&cond=gene='MCPH1) | [79648](http://www.ncbi.nlm.nih.gov/sites/entrez?db=gene&cmd=Retrieve&dopt=full_report&list_uids=79648) | Hypercholesterolemia\|LDLC levels | METABOLIC | [20602615](http://www.ncbi.nlm.nih.gov/pubmed/20602615?) |
| [MCPH1](http://geneticassociationdb.nih.gov/cgi-bin/tableview.cgi?table=diseaseview&cond=gene='MCPH1) | [79648](http://www.ncbi.nlm.nih.gov/sites/entrez?db=gene&cmd=Retrieve&dopt=full_report&list_uids=79648) | breast cancer | CANCER | [20508983](http://www.ncbi.nlm.nih.gov/pubmed/20508983?) |
| [MCPH1](http://geneticassociationdb.nih.gov/cgi-bin/tableview.cgi?table=diseaseview&cond=gene='MCPH1) | [79648](http://www.ncbi.nlm.nih.gov/sites/entrez?db=gene&cmd=Retrieve&dopt=full_report&list_uids=79648) | Microcephaly | DEVELOPMENTAL | [18204051](http://www.ncbi.nlm.nih.gov/pubmed/18204051?) |
| [ANGPT2](http://geneticassociationdb.nih.gov/cgi-bin/tableview.cgi?table=diseaseview&cond=gene='ANGPT2) | [285](http://www.ncbi.nlm.nih.gov/sites/entrez?db=gene&cmd=Retrieve&dopt=full_report&list_uids=285) | Tobacco Use Disorder | CHEMDEPENDENCY | [20379614](http://www.ncbi.nlm.nih.gov/pubmed/20379614?) |
| [ANGPT2](http://geneticassociationdb.nih.gov/cgi-bin/tableview.cgi?table=diseaseview&cond=gene='ANGPT2) | [285](http://www.ncbi.nlm.nih.gov/sites/entrez?db=gene&cmd=Retrieve&dopt=full_report&list_uids=285) | Chorioamnionitis\|Fetal Membranes, Premature Rupture\|Infection of amniotic sac and membranes | REPRODUCTION | [20673868](http://www.ncbi.nlm.nih.gov/pubmed/20673868?) |
| [ANGPT2](http://geneticassociationdb.nih.gov/cgi-bin/tableview.cgi?table=diseaseview&cond=gene='ANGPT2) | [285](http://www.ncbi.nlm.nih.gov/sites/entrez?db=gene&cmd=Retrieve&dopt=full_report&list_uids=285) | Apoplexy\|Brain Infarction\|Recurrence\|Stroke | CARDIOVASCULAR | [20599737](http://www.ncbi.nlm.nih.gov/pubmed/20599737?) |
| [ANGPT2](http://geneticassociationdb.nih.gov/cgi-bin/tableview.cgi?table=diseaseview&cond=gene='ANGPT2) | [285](http://www.ncbi.nlm.nih.gov/sites/entrez?db=gene&cmd=Retrieve&dopt=full_report&list_uids=285) | Chorioamnionitis\|Fetal Membranes, Premature Rupture\|Infection of amniotic sac and membranes\|Obstetric Labor, Premature\|Pre-Eclampsia\|Premature Birth | REPRODUCTION | [20452482](http://www.ncbi.nlm.nih.gov/pubmed/20452482?) |
| [NRG1](http://geneticassociationdb.nih.gov/cgi-bin/tableview.cgi?table=diseaseview&cond=gene='NRG1) | [3084](http://www.ncbi.nlm.nih.gov/sites/entrez?db=gene&cmd=Retrieve&dopt=full_report&list_uids=3084) | Schizophrenia | PSYCH | [19575259](http://www.ncbi.nlm.nih.gov/pubmed/19575259?) |
| [NRG1](http://geneticassociationdb.nih.gov/cgi-bin/tableview.cgi?table=diseaseview&cond=gene='NRG1) | [3084](http://www.ncbi.nlm.nih.gov/sites/entrez?db=gene&cmd=Retrieve&dopt=full_report&list_uids=3084) | Infant, Newborn, Diseases | UNKNOWN | [20472376](http://www.ncbi.nlm.nih.gov/pubmed/20472376?) |
| [NRG1](http://geneticassociationdb.nih.gov/cgi-bin/tableview.cgi?table=diseaseview&cond=gene='NRG1) | [3084](http://www.ncbi.nlm.nih.gov/sites/entrez?db=gene&cmd=Retrieve&dopt=full_report&list_uids=3084) | Schizophrenia\|bipolar disorder | PSYCH | [20435087](http://www.ncbi.nlm.nih.gov/pubmed/20435087?) |
| [NRG1](http://geneticassociationdb.nih.gov/cgi-bin/tableview.cgi?table=diseaseview&cond=gene='NRG1) | [3084](http://www.ncbi.nlm.nih.gov/sites/entrez?db=gene&cmd=Retrieve&dopt=full_report&list_uids=3084) | Tobacco Use Disorder | CHEMDEPENDENCY | [20379614](http://www.ncbi.nlm.nih.gov/pubmed/20379614?) |
| [NRG1](http://geneticassociationdb.nih.gov/cgi-bin/tableview.cgi?table=diseaseview&cond=gene='NRG1) | [3084](http://www.ncbi.nlm.nih.gov/sites/entrez?db=gene&cmd=Retrieve&dopt=full_report&list_uids=3084) | Alzheimer's disease | NEUROLOGICAL | [20182055](http://www.ncbi.nlm.nih.gov/pubmed/20182055?) |
| [NRG1](http://geneticassociationdb.nih.gov/cgi-bin/tableview.cgi?table=diseaseview&cond=gene='NRG1) | [3084](http://www.ncbi.nlm.nih.gov/sites/entrez?db=gene&cmd=Retrieve&dopt=full_report&list_uids=3084) | Schizophrenia | PSYCH | [20638435](http://www.ncbi.nlm.nih.gov/pubmed/20638435?) |
| [NRG1](http://geneticassociationdb.nih.gov/cgi-bin/tableview.cgi?table=diseaseview&cond=gene='NRG1) | [3084](http://www.ncbi.nlm.nih.gov/sites/entrez?db=gene&cmd=Retrieve&dopt=full_report&list_uids=3084) | Type 2 Diabetes\| edema \| rosiglitazone | PHARMACOGENOMIC | [20628086](http://www.ncbi.nlm.nih.gov/pubmed/20628086?) |
| [NRG1](http://geneticassociationdb.nih.gov/cgi-bin/tableview.cgi?table=diseaseview&cond=gene='NRG1) | [3084](http://www.ncbi.nlm.nih.gov/sites/entrez?db=gene&cmd=Retrieve&dopt=full_report&list_uids=3084) | Schizophrenia | PSYCH | [20526724](http://www.ncbi.nlm.nih.gov/pubmed/20526724?) |
| [NRG1](http://geneticassociationdb.nih.gov/cgi-bin/tableview.cgi?table=diseaseview&cond=gene='NRG1) | [3084](http://www.ncbi.nlm.nih.gov/sites/entrez?db=gene&cmd=Retrieve&dopt=full_report&list_uids=3084) | Marijuana Abuse\|Psychoses, Substance-Induced | CHEMDEPENDENCY | [21041608](http://www.ncbi.nlm.nih.gov/pubmed/21041608?) |
| [NRG1](http://geneticassociationdb.nih.gov/cgi-bin/tableview.cgi?table=diseaseview&cond=gene='NRG1) | [3084](http://www.ncbi.nlm.nih.gov/sites/entrez?db=gene&cmd=Retrieve&dopt=full_report&list_uids=3084) | Schizophrenia | PSYCH | [20921115](http://www.ncbi.nlm.nih.gov/pubmed/20921115?) |
| [NRG1](http://geneticassociationdb.nih.gov/cgi-bin/tableview.cgi?table=diseaseview&cond=gene='NRG1) | [3084](http://www.ncbi.nlm.nih.gov/sites/entrez?db=gene&cmd=Retrieve&dopt=full_report&list_uids=3084) | longevity | AGING | [20800603](http://www.ncbi.nlm.nih.gov/pubmed/20800603?) |
| [NRG1](http://geneticassociationdb.nih.gov/cgi-bin/tableview.cgi?table=diseaseview&cond=gene='NRG1) | [3084](http://www.ncbi.nlm.nih.gov/sites/entrez?db=gene&cmd=Retrieve&dopt=full_report&list_uids=3084) | Schizophrenia | PSYCH | [18282690](http://www.ncbi.nlm.nih.gov/pubmed/18282690?) |
| [NRG1](http://geneticassociationdb.nih.gov/cgi-bin/tableview.cgi?table=diseaseview&cond=gene='NRG1) | [3084](http://www.ncbi.nlm.nih.gov/sites/entrez?db=gene&cmd=Retrieve&dopt=full_report&list_uids=3084) | Schizophrenia | PSYCH | [18198266](http://www.ncbi.nlm.nih.gov/pubmed/18198266?) |
| [NRG1](http://geneticassociationdb.nih.gov/cgi-bin/tableview.cgi?table=diseaseview&cond=gene='NRG1) | [3084](http://www.ncbi.nlm.nih.gov/sites/entrez?db=gene&cmd=Retrieve&dopt=full_report&list_uids=3084) | Schizophrenia | PSYCH | [18032396](http://www.ncbi.nlm.nih.gov/pubmed/18032396?) |
| [NRG1](http://geneticassociationdb.nih.gov/cgi-bin/tableview.cgi?table=diseaseview&cond=gene='NRG1) | [3084](http://www.ncbi.nlm.nih.gov/sites/entrez?db=gene&cmd=Retrieve&dopt=full_report&list_uids=3084) | prepulse inhibition | UNKNOWN | [17631867](http://www.ncbi.nlm.nih.gov/pubmed/17631867?) |
| [NRG1](http://geneticassociationdb.nih.gov/cgi-bin/tableview.cgi?table=diseaseview&cond=gene='NRG1) | [3084](http://www.ncbi.nlm.nih.gov/sites/entrez?db=gene&cmd=Retrieve&dopt=full_report&list_uids=3084) | Hippocampal Atrophy | UNKNOWN | [18291420](http://www.ncbi.nlm.nih.gov/pubmed/18291420?) |
| [NRG1](http://geneticassociationdb.nih.gov/cgi-bin/tableview.cgi?table=diseaseview&cond=gene='NRG1) | [3084](http://www.ncbi.nlm.nih.gov/sites/entrez?db=gene&cmd=Retrieve&dopt=full_report&list_uids=3084) | Schizophrenia | PSYCH | [18286587](http://www.ncbi.nlm.nih.gov/pubmed/18286587?) |
| [PHACTR2](http://geneticassociationdb.nih.gov/cgi-bin/tableview.cgi?table=diseaseview&cond=gene='PHACTR2) | [9749](http://www.ncbi.nlm.nih.gov/sites/entrez?db=gene&cmd=Retrieve&dopt=full_report&list_uids=9749) | Parkinson's disease | NEUROLOGICAL | [19429005](http://www.ncbi.nlm.nih.gov/pubmed/19429005?) |
| [PHACTR2](http://geneticassociationdb.nih.gov/cgi-bin/tableview.cgi?table=diseaseview&cond=gene='PHACTR2) | [9749](http://www.ncbi.nlm.nih.gov/sites/entrez?db=gene&cmd=Retrieve&dopt=full_report&list_uids=9749) | Alzheimer's disease | NEUROLOGICAL | [19141999](http://www.ncbi.nlm.nih.gov/pubmed/19141999?) |
| [PHACTR2](http://geneticassociationdb.nih.gov/cgi-bin/tableview.cgi?table=diseaseview&cond=gene='PHACTR2) | [9749](http://www.ncbi.nlm.nih.gov/sites/entrez?db=gene&cmd=Retrieve&dopt=full_report&list_uids=9749) | Type 2 Diabetes\| edema \| rosiglitazone | PHARMACOGENOMIC | [20628086](http://www.ncbi.nlm.nih.gov/pubmed/20628086?) |
| [PHACTR2](http://geneticassociationdb.nih.gov/cgi-bin/tableview.cgi?table=diseaseview&cond=gene='PHACTR2) | [9749](http://www.ncbi.nlm.nih.gov/sites/entrez?db=gene&cmd=Retrieve&dopt=full_report&list_uids=9749) | Multiple Sclerosis | IMMUNE | [20546594](http://www.ncbi.nlm.nih.gov/pubmed/20546594?) |
| [PHACTR2](http://geneticassociationdb.nih.gov/cgi-bin/tableview.cgi?table=diseaseview&cond=gene='PHACTR2) | [9749](http://www.ncbi.nlm.nih.gov/sites/entrez?db=gene&cmd=Retrieve&dopt=full_report&list_uids=9749) | Tobacco Use Disorder | CHEMDEPENDENCY | [20379614](http://www.ncbi.nlm.nih.gov/pubmed/20379614?) |
| [GPR109A](http://geneticassociationdb.nih.gov/cgi-bin/tableview.cgi?table=diseaseview&cond=gene='GPR109A) | [338442](http://www.ncbi.nlm.nih.gov/sites/entrez?db=gene&cmd=Retrieve&dopt=full_report&list_uids=338442) | schizophrenia \| bipolar disorder | PSYCH | [19502010](http://www.ncbi.nlm.nih.gov/pubmed/19502010?) |
| [CSMD1](http://geneticassociationdb.nih.gov/cgi-bin/tableview.cgi?table=diseaseview&cond=gene='CSMD1) | [64478](http://www.ncbi.nlm.nih.gov/sites/entrez?db=gene&cmd=Retrieve&dopt=full_report&list_uids=64478) | Tobacco Use Disorder | CHEMDEPENDENCY | [18519826](http://www.ncbi.nlm.nih.gov/pubmed/18519826?) |
| [CSMD1](http://geneticassociationdb.nih.gov/cgi-bin/tableview.cgi?table=diseaseview&cond=gene='CSMD1) | [64478](http://www.ncbi.nlm.nih.gov/sites/entrez?db=gene&cmd=Retrieve&dopt=full_report&list_uids=64478) | Chromosomal Instability\|Cystadenocarcinoma, Serous\|Ovarian Neoplasms | CANCER | [19383911](http://www.ncbi.nlm.nih.gov/pubmed/19383911?) |
| [CSMD1](http://geneticassociationdb.nih.gov/cgi-bin/tableview.cgi?table=diseaseview&cond=gene='CSMD1) | [64478](http://www.ncbi.nlm.nih.gov/sites/entrez?db=gene&cmd=Retrieve&dopt=full_report&list_uids=64478) | Celiac Disease\| | IMMUNE | [19240061](http://www.ncbi.nlm.nih.gov/pubmed/19240061?) |
| [CSMD1](http://geneticassociationdb.nih.gov/cgi-bin/tableview.cgi?table=diseaseview&cond=gene='CSMD1) | [64478](http://www.ncbi.nlm.nih.gov/sites/entrez?db=gene&cmd=Retrieve&dopt=full_report&list_uids=64478) | Mucocutaneous Lymph Node Syndrome | IMMUNE | [19132087](http://www.ncbi.nlm.nih.gov/pubmed/19132087?) |
| [CSMD1](http://geneticassociationdb.nih.gov/cgi-bin/tableview.cgi?table=diseaseview&cond=gene='CSMD1) | [64478](http://www.ncbi.nlm.nih.gov/sites/entrez?db=gene&cmd=Retrieve&dopt=full_report&list_uids=64478) | hypertension | CARDIOVASCULAR | [19960030](http://www.ncbi.nlm.nih.gov/pubmed/19960030?) |
| [CSMD1](http://geneticassociationdb.nih.gov/cgi-bin/tableview.cgi?table=diseaseview&cond=gene='CSMD1) | [64478](http://www.ncbi.nlm.nih.gov/sites/entrez?db=gene&cmd=Retrieve&dopt=full_report&list_uids=64478) | multiple sclerosis | IMMUNE | [19010793](http://www.ncbi.nlm.nih.gov/pubmed/19010793?) |
| [CSMD1](http://geneticassociationdb.nih.gov/cgi-bin/tableview.cgi?table=diseaseview&cond=gene='CSMD1) | [64478](http://www.ncbi.nlm.nih.gov/sites/entrez?db=gene&cmd=Retrieve&dopt=full_report&list_uids=64478) | smoking cessation | CHEMDEPENDENCY | [20235792](http://www.ncbi.nlm.nih.gov/pubmed/20235792?) |
| [CSMD1](http://geneticassociationdb.nih.gov/cgi-bin/tableview.cgi?table=diseaseview&cond=gene='CSMD1) | [64478](http://www.ncbi.nlm.nih.gov/sites/entrez?db=gene&cmd=Retrieve&dopt=full_report&list_uids=64478) | smoking cessation | CHEMDEPENDENCY | [20235792](http://www.ncbi.nlm.nih.gov/pubmed/20235792?) |
| [CSMD1](http://geneticassociationdb.nih.gov/cgi-bin/tableview.cgi?table=diseaseview&cond=gene='CSMD1) | [64478](http://www.ncbi.nlm.nih.gov/sites/entrez?db=gene&cmd=Retrieve&dopt=full_report&list_uids=64478) | Psoriasis | IMMUNE | [20953187](http://www.ncbi.nlm.nih.gov/pubmed/20953187?) |
| [CSMD1](http://geneticassociationdb.nih.gov/cgi-bin/tableview.cgi?table=diseaseview&cond=gene='CSMD1) | [64478](http://www.ncbi.nlm.nih.gov/sites/entrez?db=gene&cmd=Retrieve&dopt=full_report&list_uids=64478) | Peripheral Vascular Diseases | CARDIOVASCULAR | [20610895](http://www.ncbi.nlm.nih.gov/pubmed/20610895?) |
| [CSMD1](http://geneticassociationdb.nih.gov/cgi-bin/tableview.cgi?table=diseaseview&cond=gene='CSMD1) | [64478](http://www.ncbi.nlm.nih.gov/sites/entrez?db=gene&cmd=Retrieve&dopt=full_report&list_uids=64478) | Tobacco Use Disorder | CHEMDEPENDENCY | [20379614](http://www.ncbi.nlm.nih.gov/pubmed/20379614?) |
| [CAMTA1](http://geneticassociationdb.nih.gov/cgi-bin/tableview.cgi?table=diseaseview&cond=gene='CAMTA1) | [23261](http://www.ncbi.nlm.nih.gov/sites/entrez?db=gene&cmd=Retrieve&dopt=full_report&list_uids=23261) | memory disturbance | PSYCH | [17470457](http://www.ncbi.nlm.nih.gov/pubmed/17470457?) |
| [CAMTA1](http://geneticassociationdb.nih.gov/cgi-bin/tableview.cgi?table=diseaseview&cond=gene='CAMTA1) | [23261](http://www.ncbi.nlm.nih.gov/sites/entrez?db=gene&cmd=Retrieve&dopt=full_report&list_uids=23261) | hypertension | CARDIOVASCULAR | [19851296](http://www.ncbi.nlm.nih.gov/pubmed/19851296?) |
| [CAMTA1](http://geneticassociationdb.nih.gov/cgi-bin/tableview.cgi?table=diseaseview&cond=gene='CAMTA1) | [23261](http://www.ncbi.nlm.nih.gov/sites/entrez?db=gene&cmd=Retrieve&dopt=full_report&list_uids=23261) | Coronary Disease | CARDIOVASCULAR | [19336475](http://www.ncbi.nlm.nih.gov/pubmed/19336475?) |
| [CAMTA1](http://geneticassociationdb.nih.gov/cgi-bin/tableview.cgi?table=diseaseview&cond=gene='CAMTA1) | [23261](http://www.ncbi.nlm.nih.gov/sites/entrez?db=gene&cmd=Retrieve&dopt=full_report&list_uids=23261) | Type 2 Diabetes\| edema \| rosiglitazone | PHARMACOGENOMIC | [20628086](http://www.ncbi.nlm.nih.gov/pubmed/20628086?) |
| [CAMTA1](http://geneticassociationdb.nih.gov/cgi-bin/tableview.cgi?table=diseaseview&cond=gene='CAMTA1) | [23261](http://www.ncbi.nlm.nih.gov/sites/entrez?db=gene&cmd=Retrieve&dopt=full_report&list_uids=23261) | Tobacco Use Disorder | CHEMDEPENDENCY | [20379614](http://www.ncbi.nlm.nih.gov/pubmed/20379614?) |
| [CAMTA1](http://geneticassociationdb.nih.gov/cgi-bin/tableview.cgi?table=diseaseview&cond=gene='CAMTA1) | [23261](http://www.ncbi.nlm.nih.gov/sites/entrez?db=gene&cmd=Retrieve&dopt=full_report&list_uids=23261) | Apoplexy\|Cerebral Hemorrhage\|Cerebral Hemorrhages\|Intracranial Hemorrhages\|Stroke\|Subarachnoid Hemorrhage | CARDIOVASCULAR | [20198315](http://www.ncbi.nlm.nih.gov/pubmed/20198315?) |
| [CAMTA1](http://geneticassociationdb.nih.gov/cgi-bin/tableview.cgi?table=diseaseview&cond=gene='CAMTA1) | [23261](http://www.ncbi.nlm.nih.gov/sites/entrez?db=gene&cmd=Retrieve&dopt=full_report&list_uids=23261) | Type 2 diabetes | METABOLIC | [18210030](http://www.ncbi.nlm.nih.gov/pubmed/18210030?) |
| [AGBL4](http://geneticassociationdb.nih.gov/cgi-bin/tableview.cgi?table=diseaseview&cond=gene='AGBL4) | [84871](http://www.ncbi.nlm.nih.gov/sites/entrez?db=gene&cmd=Retrieve&dopt=full_report&list_uids=84871) | Celiac Disease\| | IMMUNE | [19240061](http://www.ncbi.nlm.nih.gov/pubmed/19240061?) |
| [AGBL4](http://geneticassociationdb.nih.gov/cgi-bin/tableview.cgi?table=diseaseview&cond=gene='AGBL4) | [84871](http://www.ncbi.nlm.nih.gov/sites/entrez?db=gene&cmd=Retrieve&dopt=full_report&list_uids=84871) | Tobacco Use Disorder | CHEMDEPENDENCY | [20379614](http://www.ncbi.nlm.nih.gov/pubmed/20379614?) |
| [PACRG](http://geneticassociationdb.nih.gov/cgi-bin/tableview.cgi?table=diseaseview&cond=gene='PACRG) | [135138](http://www.ncbi.nlm.nih.gov/sites/entrez?db=gene&cmd=Retrieve&dopt=full_report&list_uids=135138) | leprosy | INFECTION | [14737177](http://www.ncbi.nlm.nih.gov/pubmed/14737177?) |
| [PACRG](http://geneticassociationdb.nih.gov/cgi-bin/tableview.cgi?table=diseaseview&cond=gene='PACRG) | [135138](http://www.ncbi.nlm.nih.gov/sites/entrez?db=gene&cmd=Retrieve&dopt=full_report&list_uids=135138) | leprosy | INFECTION | [16391553](http://www.ncbi.nlm.nih.gov/pubmed/16391553?) |
| [PACRG](http://geneticassociationdb.nih.gov/cgi-bin/tableview.cgi?table=diseaseview&cond=gene='PACRG) | [135138](http://www.ncbi.nlm.nih.gov/sites/entrez?db=gene&cmd=Retrieve&dopt=full_report&list_uids=135138) | Parkinson's disease | NEUROLOGICAL | [15925106](http://www.ncbi.nlm.nih.gov/pubmed/15925106?) |
| [PACRG](http://geneticassociationdb.nih.gov/cgi-bin/tableview.cgi?table=diseaseview&cond=gene='PACRG) | [135138](http://www.ncbi.nlm.nih.gov/sites/entrez?db=gene&cmd=Retrieve&dopt=full_report&list_uids=135138) | leprosy | INFECTION | [14737177](http://www.ncbi.nlm.nih.gov/pubmed/14737177?) |
| [PACRG](http://geneticassociationdb.nih.gov/cgi-bin/tableview.cgi?table=diseaseview&cond=gene='PACRG) | [135138](http://www.ncbi.nlm.nih.gov/sites/entrez?db=gene&cmd=Retrieve&dopt=full_report&list_uids=135138) | Parkinson's disease | NEUROLOGICAL | [19196541](http://www.ncbi.nlm.nih.gov/pubmed/19196541?) |
| [PACRG](http://geneticassociationdb.nih.gov/cgi-bin/tableview.cgi?table=diseaseview&cond=gene='PACRG) | [135138](http://www.ncbi.nlm.nih.gov/sites/entrez?db=gene&cmd=Retrieve&dopt=full_report&list_uids=135138) | Tuberculosis | INFECTION | [19723394](http://www.ncbi.nlm.nih.gov/pubmed/19723394?) |
| [PACRG](http://geneticassociationdb.nih.gov/cgi-bin/tableview.cgi?table=diseaseview&cond=gene='PACRG) | [135138](http://www.ncbi.nlm.nih.gov/sites/entrez?db=gene&cmd=Retrieve&dopt=full_report&list_uids=135138) | male infertility | REPRODUCTION | [19268936](http://www.ncbi.nlm.nih.gov/pubmed/19268936?) |
| [PACRG](http://geneticassociationdb.nih.gov/cgi-bin/tableview.cgi?table=diseaseview&cond=gene='PACRG) | [135138](http://www.ncbi.nlm.nih.gov/sites/entrez?db=gene&cmd=Retrieve&dopt=full_report&list_uids=135138) | Acquired Immunodeficiency Syndrome\|Disease Progression | INFECTION | [20877624](http://www.ncbi.nlm.nih.gov/pubmed/20877624?) |
| [PACRG](http://geneticassociationdb.nih.gov/cgi-bin/tableview.cgi?table=diseaseview&cond=gene='PACRG) | [135138](http://www.ncbi.nlm.nih.gov/sites/entrez?db=gene&cmd=Retrieve&dopt=full_report&list_uids=135138) | Tobacco Use Disorder | CHEMDEPENDENCY | [20379614](http://www.ncbi.nlm.nih.gov/pubmed/20379614?) |
| [CPNE4](http://geneticassociationdb.nih.gov/cgi-bin/tableview.cgi?table=diseaseview&cond=gene='CPNE4) | [131034](http://www.ncbi.nlm.nih.gov/sites/entrez?db=gene&cmd=Retrieve&dopt=full_report&list_uids=131034) | Tobacco Use Disorder | CHEMDEPENDENCY | [20379614](http://www.ncbi.nlm.nih.gov/pubmed/20379614?) |
| [TTC29](http://geneticassociationdb.nih.gov/cgi-bin/tableview.cgi?table=diseaseview&cond=gene='TTC29) | [83894](http://www.ncbi.nlm.nih.gov/sites/entrez?db=gene&cmd=Retrieve&dopt=full_report&list_uids=83894) | Tobacco Use Disorder | CHEMDEPENDENCY | [20379614](http://www.ncbi.nlm.nih.gov/pubmed/20379614?) |
| [GPR109A](http://geneticassociationdb.nih.gov/cgi-bin/tableview.cgi?table=diseaseview&cond=gene='GPR109A) | [338442](http://www.ncbi.nlm.nih.gov/sites/entrez?db=gene&cmd=Retrieve&dopt=full_report&list_uids=338442) | schizophrenia \| bipolar disorder | PSYCH | [19502010](http://www.ncbi.nlm.nih.gov/pubmed/19502010?) |
| [DEPDC6](http://geneticassociationdb.nih.gov/cgi-bin/tableview.cgi?table=diseaseview&cond=gene='DEPDC6) | [64798](http://www.ncbi.nlm.nih.gov/sites/entrez?db=gene&cmd=Retrieve&dopt=full_report&list_uids=64798) | Tobacco Use Disorder | CHEMDEPENDENCY | [20379614](http://www.ncbi.nlm.nih.gov/pubmed/20379614?) |
| [CSMD1](http://geneticassociationdb.nih.gov/cgi-bin/tableview.cgi?table=diseaseview&cond=gene='CSMD1) | [64478](http://www.ncbi.nlm.nih.gov/sites/entrez?db=gene&cmd=Retrieve&dopt=full_report&list_uids=64478) | Tobacco Use Disorder | CHEMDEPENDENCY | [18519826](http://www.ncbi.nlm.nih.gov/pubmed/18519826?) |
| [CSMD1](http://geneticassociationdb.nih.gov/cgi-bin/tableview.cgi?table=diseaseview&cond=gene='CSMD1) | [64478](http://www.ncbi.nlm.nih.gov/sites/entrez?db=gene&cmd=Retrieve&dopt=full_report&list_uids=64478) | Chromosomal Instability\|Cystadenocarcinoma, Serous\|Ovarian Neoplasms | CANCER | [19383911](http://www.ncbi.nlm.nih.gov/pubmed/19383911?) |
| [CSMD1](http://geneticassociationdb.nih.gov/cgi-bin/tableview.cgi?table=diseaseview&cond=gene='CSMD1) | [64478](http://www.ncbi.nlm.nih.gov/sites/entrez?db=gene&cmd=Retrieve&dopt=full_report&list_uids=64478) | Celiac Disease\| | IMMUNE | [19240061](http://www.ncbi.nlm.nih.gov/pubmed/19240061?) |
| [CSMD1](http://geneticassociationdb.nih.gov/cgi-bin/tableview.cgi?table=diseaseview&cond=gene='CSMD1) | [64478](http://www.ncbi.nlm.nih.gov/sites/entrez?db=gene&cmd=Retrieve&dopt=full_report&list_uids=64478) | Mucocutaneous Lymph Node Syndrome | IMMUNE | [19132087](http://www.ncbi.nlm.nih.gov/pubmed/19132087?) |
| [CSMD1](http://geneticassociationdb.nih.gov/cgi-bin/tableview.cgi?table=diseaseview&cond=gene='CSMD1) | [64478](http://www.ncbi.nlm.nih.gov/sites/entrez?db=gene&cmd=Retrieve&dopt=full_report&list_uids=64478) | hypertension | CARDIOVASCULAR | [19960030](http://www.ncbi.nlm.nih.gov/pubmed/19960030?) |
| [CSMD1](http://geneticassociationdb.nih.gov/cgi-bin/tableview.cgi?table=diseaseview&cond=gene='CSMD1) | [64478](http://www.ncbi.nlm.nih.gov/sites/entrez?db=gene&cmd=Retrieve&dopt=full_report&list_uids=64478) | multiple sclerosis | IMMUNE | [19010793](http://www.ncbi.nlm.nih.gov/pubmed/19010793?) |
| [CSMD1](http://geneticassociationdb.nih.gov/cgi-bin/tableview.cgi?table=diseaseview&cond=gene='CSMD1) | [64478](http://www.ncbi.nlm.nih.gov/sites/entrez?db=gene&cmd=Retrieve&dopt=full_report&list_uids=64478) | smoking cessation | CHEMDEPENDENCY | [20235792](http://www.ncbi.nlm.nih.gov/pubmed/20235792?) |
| [CSMD1](http://geneticassociationdb.nih.gov/cgi-bin/tableview.cgi?table=diseaseview&cond=gene='CSMD1) | [64478](http://www.ncbi.nlm.nih.gov/sites/entrez?db=gene&cmd=Retrieve&dopt=full_report&list_uids=64478) | smoking cessation | CHEMDEPENDENCY | [20235792](http://www.ncbi.nlm.nih.gov/pubmed/20235792?) |
| [CSMD1](http://geneticassociationdb.nih.gov/cgi-bin/tableview.cgi?table=diseaseview&cond=gene='CSMD1) | [64478](http://www.ncbi.nlm.nih.gov/sites/entrez?db=gene&cmd=Retrieve&dopt=full_report&list_uids=64478) | Psoriasis | IMMUNE | [20953187](http://www.ncbi.nlm.nih.gov/pubmed/20953187?) |
| [CSMD1](http://geneticassociationdb.nih.gov/cgi-bin/tableview.cgi?table=diseaseview&cond=gene='CSMD1) | [64478](http://www.ncbi.nlm.nih.gov/sites/entrez?db=gene&cmd=Retrieve&dopt=full_report&list_uids=64478) | Peripheral Vascular Diseases | CARDIOVASCULAR | [20610895](http://www.ncbi.nlm.nih.gov/pubmed/20610895?) |
| [CSMD1](http://geneticassociationdb.nih.gov/cgi-bin/tableview.cgi?table=diseaseview&cond=gene='CSMD1) | [64478](http://www.ncbi.nlm.nih.gov/sites/entrez?db=gene&cmd=Retrieve&dopt=full_report&list_uids=64478) | Tobacco Use Disorder | CHEMDEPENDENCY | [20379614](http://www.ncbi.nlm.nih.gov/pubmed/20379614?) |
| [SESTD1](http://geneticassociationdb.nih.gov/cgi-bin/tableview.cgi?table=diseaseview&cond=gene='SESTD1) | [91404](http://www.ncbi.nlm.nih.gov/sites/entrez?db=gene&cmd=Retrieve&dopt=full_report&list_uids=91404) | Celiac Disease\| | IMMUNE | [19240061](http://www.ncbi.nlm.nih.gov/pubmed/19240061?) |
| [SESTD1](http://geneticassociationdb.nih.gov/cgi-bin/tableview.cgi?table=diseaseview&cond=gene='SESTD1) | [91404](http://www.ncbi.nlm.nih.gov/sites/entrez?db=gene&cmd=Retrieve&dopt=full_report&list_uids=91404) | HIV Infections\|[X]Human immunodeficiency virus disease | INFECTION | [21083371](http://www.ncbi.nlm.nih.gov/pubmed/21083371?) |
| [SESTD1](http://geneticassociationdb.nih.gov/cgi-bin/tableview.cgi?table=diseaseview&cond=gene='SESTD1) | [91404](http://www.ncbi.nlm.nih.gov/sites/entrez?db=gene&cmd=Retrieve&dopt=full_report&list_uids=91404) | Tobacco Use Disorder | CHEMDEPENDENCY | [20379614](http://www.ncbi.nlm.nih.gov/pubmed/20379614?) |
| [LRP1B](http://geneticassociationdb.nih.gov/cgi-bin/tableview.cgi?table=diseaseview&cond=gene='LRP1B) | [53353](http://www.ncbi.nlm.nih.gov/sites/entrez?db=gene&cmd=Retrieve&dopt=full_report&list_uids=53353) | cognitive ability | NEUROLOGICAL | [19367585](http://www.ncbi.nlm.nih.gov/pubmed/19367585?) |
| [LRP1B](http://geneticassociationdb.nih.gov/cgi-bin/tableview.cgi?table=diseaseview&cond=gene='LRP1B) | [53353](http://www.ncbi.nlm.nih.gov/sites/entrez?db=gene&cmd=Retrieve&dopt=full_report&list_uids=53353) | Aging | AGING | [19367585](http://www.ncbi.nlm.nih.gov/pubmed/19367585?) |
| [LRP1B](http://geneticassociationdb.nih.gov/cgi-bin/tableview.cgi?table=diseaseview&cond=gene='LRP1B) | [53353](http://www.ncbi.nlm.nih.gov/sites/entrez?db=gene&cmd=Retrieve&dopt=full_report&list_uids=53353) | Tobacco Use Disorder | CHEMDEPENDENCY | [20379614](http://www.ncbi.nlm.nih.gov/pubmed/20379614?) |
| [KLF7](http://geneticassociationdb.nih.gov/cgi-bin/tableview.cgi?table=diseaseview&cond=gene='KLF7) | [8609](http://www.ncbi.nlm.nih.gov/sites/entrez?db=gene&cmd=Retrieve&dopt=full_report&list_uids=8609) | diabetes, type 2 | METABOLIC | [15937668](http://www.ncbi.nlm.nih.gov/pubmed/15937668?) |
| [KLF7](http://geneticassociationdb.nih.gov/cgi-bin/tableview.cgi?table=diseaseview&cond=gene='KLF7) | [8609](http://www.ncbi.nlm.nih.gov/sites/entrez?db=gene&cmd=Retrieve&dopt=full_report&list_uids=8609) | Body Weight\|Diabetes Mellitus, Type 2\|Obesity\|Overweight | METABOLIC | [19147600](http://www.ncbi.nlm.nih.gov/pubmed/19147600?) |
| [MAGI2](http://geneticassociationdb.nih.gov/cgi-bin/tableview.cgi?table=diseaseview&cond=gene='MAGI2) | [9863](http://www.ncbi.nlm.nih.gov/sites/entrez?db=gene&cmd=Retrieve&dopt=full_report&list_uids=9863) | several psychiatric disorders | PSYCH | [19086053](http://www.ncbi.nlm.nih.gov/pubmed/19086053?) |
| [MAGI2](http://geneticassociationdb.nih.gov/cgi-bin/tableview.cgi?table=diseaseview&cond=gene='MAGI2) | [9863](http://www.ncbi.nlm.nih.gov/sites/entrez?db=gene&cmd=Retrieve&dopt=full_report&list_uids=9863) | inflammatory bowel disease | IMMUNE | [18720471](http://www.ncbi.nlm.nih.gov/pubmed/18720471?) |
| [MAGI2](http://geneticassociationdb.nih.gov/cgi-bin/tableview.cgi?table=diseaseview&cond=gene='MAGI2) | [9863](http://www.ncbi.nlm.nih.gov/sites/entrez?db=gene&cmd=Retrieve&dopt=full_report&list_uids=9863) | Celiac Disease\| | IMMUNE | [19240061](http://www.ncbi.nlm.nih.gov/pubmed/19240061?) |
| [MAGI2](http://geneticassociationdb.nih.gov/cgi-bin/tableview.cgi?table=diseaseview&cond=gene='MAGI2) | [9863](http://www.ncbi.nlm.nih.gov/sites/entrez?db=gene&cmd=Retrieve&dopt=full_report&list_uids=9863) | hippocampal atrophy | NEUROLOGICAL | [19668339](http://www.ncbi.nlm.nih.gov/pubmed/19668339?) |
| [MAGI2](http://geneticassociationdb.nih.gov/cgi-bin/tableview.cgi?table=diseaseview&cond=gene='MAGI2) | [9863](http://www.ncbi.nlm.nih.gov/sites/entrez?db=gene&cmd=Retrieve&dopt=full_report&list_uids=9863) | Type 2 Diabetes\| edema \| rosiglitazone | PHARMACOGENOMIC | [20628086](http://www.ncbi.nlm.nih.gov/pubmed/20628086?) |
| [MAGI2](http://geneticassociationdb.nih.gov/cgi-bin/tableview.cgi?table=diseaseview&cond=gene='MAGI2) | [9863](http://www.ncbi.nlm.nih.gov/sites/entrez?db=gene&cmd=Retrieve&dopt=full_report&list_uids=9863) | Tobacco Use Disorder | CHEMDEPENDENCY | [20379614](http://www.ncbi.nlm.nih.gov/pubmed/20379614?) |
| [MAGI2](http://geneticassociationdb.nih.gov/cgi-bin/tableview.cgi?table=diseaseview&cond=gene='MAGI2) | [9863](http://www.ncbi.nlm.nih.gov/sites/entrez?db=gene&cmd=Retrieve&dopt=full_report&list_uids=9863) | Celiac Disease\|Down Syndrome | IMMUNE | [20096742](http://www.ncbi.nlm.nih.gov/pubmed/20096742?) |
| [MAGI2](http://geneticassociationdb.nih.gov/cgi-bin/tableview.cgi?table=diseaseview&cond=gene='MAGI2) | [9863](http://www.ncbi.nlm.nih.gov/sites/entrez?db=gene&cmd=Retrieve&dopt=full_report&list_uids=9863) | Celiac Disease\|Colitis, Ulcerative | IMMUNE | [17989107](http://www.ncbi.nlm.nih.gov/pubmed/17989107?) |
| [BARX2](http://geneticassociationdb.nih.gov/cgi-bin/tableview.cgi?table=diseaseview&cond=gene='BARX2) | [8538](http://www.ncbi.nlm.nih.gov/sites/entrez?db=gene&cmd=Retrieve&dopt=full_report&list_uids=8538) | Cleft Lip\|Cleft Palate | DEVELOPMENTAL | [20634891](http://www.ncbi.nlm.nih.gov/pubmed/20634891?) |
| [ATP2C2](http://geneticassociationdb.nih.gov/cgi-bin/tableview.cgi?table=diseaseview&cond=gene='ATP2C2) | [9914](http://www.ncbi.nlm.nih.gov/sites/entrez?db=gene&cmd=Retrieve&dopt=full_report&list_uids=9914) | Migraine without Aura | NEUROLOGICAL | [18676988](http://www.ncbi.nlm.nih.gov/pubmed/18676988?) |
| [ATP2C2](http://geneticassociationdb.nih.gov/cgi-bin/tableview.cgi?table=diseaseview&cond=gene='ATP2C2) | [9914](http://www.ncbi.nlm.nih.gov/sites/entrez?db=gene&cmd=Retrieve&dopt=full_report&list_uids=9914) | ADHD \| attention-deficit hyperactivity disorder | PSYCH | [18839057](http://www.ncbi.nlm.nih.gov/pubmed/18839057?) |
| [ATP2C2](http://geneticassociationdb.nih.gov/cgi-bin/tableview.cgi?table=diseaseview&cond=gene='ATP2C2) | [9914](http://www.ncbi.nlm.nih.gov/sites/entrez?db=gene&cmd=Retrieve&dopt=full_report&list_uids=9914) | Tobacco Use Disorder | CHEMDEPENDENCY | [20379614](http://www.ncbi.nlm.nih.gov/pubmed/20379614?) |
| [CD247](http://geneticassociationdb.nih.gov/cgi-bin/tableview.cgi?table=diseaseview&cond=gene='CD247) | [919](http://www.ncbi.nlm.nih.gov/sites/entrez?db=gene&cmd=Retrieve&dopt=full_report&list_uids=919) | Osteoporosis | METABOLIC | [19064610](http://www.ncbi.nlm.nih.gov/pubmed/19064610?) |
| [CD247](http://geneticassociationdb.nih.gov/cgi-bin/tableview.cgi?table=diseaseview&cond=gene='CD247) | [919](http://www.ncbi.nlm.nih.gov/sites/entrez?db=gene&cmd=Retrieve&dopt=full_report&list_uids=919) | Arthritis, Rheumatoid\| | IMMUNE | [19898481](http://www.ncbi.nlm.nih.gov/pubmed/19898481?) |
| [CD247](http://geneticassociationdb.nih.gov/cgi-bin/tableview.cgi?table=diseaseview&cond=gene='CD247) | [919](http://www.ncbi.nlm.nih.gov/sites/entrez?db=gene&cmd=Retrieve&dopt=full_report&list_uids=919) | Hypertension | CARDIOVASCULAR | [19536175](http://www.ncbi.nlm.nih.gov/pubmed/19536175?) |
| [CD247](http://geneticassociationdb.nih.gov/cgi-bin/tableview.cgi?table=diseaseview&cond=gene='CD247) | [919](http://www.ncbi.nlm.nih.gov/sites/entrez?db=gene&cmd=Retrieve&dopt=full_report&list_uids=919) | Lupus Erythematosus, Systemic | IMMUNE | [19422667](http://www.ncbi.nlm.nih.gov/pubmed/19422667?) |
| [CD247](http://geneticassociationdb.nih.gov/cgi-bin/tableview.cgi?table=diseaseview&cond=gene='CD247) | [919](http://www.ncbi.nlm.nih.gov/sites/entrez?db=gene&cmd=Retrieve&dopt=full_report&list_uids=919) | Celiac disease | IMMUNE | [20190752](http://www.ncbi.nlm.nih.gov/pubmed/20190752?) |
| [CD247](http://geneticassociationdb.nih.gov/cgi-bin/tableview.cgi?table=diseaseview&cond=gene='CD247) | [919](http://www.ncbi.nlm.nih.gov/sites/entrez?db=gene&cmd=Retrieve&dopt=full_report&list_uids=919) | Scleroderma, Systemic\|Systemic Scleroderma | IMMUNE | [20383147](http://www.ncbi.nlm.nih.gov/pubmed/20383147?) |
| [CD247](http://geneticassociationdb.nih.gov/cgi-bin/tableview.cgi?table=diseaseview&cond=gene='CD247) | [919](http://www.ncbi.nlm.nih.gov/sites/entrez?db=gene&cmd=Retrieve&dopt=full_report&list_uids=919) | Lupus Erythematosus, Systemic\|Systemic lupus erythematosus | IMMUNE | [18178846](http://www.ncbi.nlm.nih.gov/pubmed/18178846?) |
| [CD247](http://geneticassociationdb.nih.gov/cgi-bin/tableview.cgi?table=diseaseview&cond=gene='CD247) | [919](http://www.ncbi.nlm.nih.gov/sites/entrez?db=gene&cmd=Retrieve&dopt=full_report&list_uids=919) | Lupus Erythematosus, Systemic\|Systemic lupus erythematosus | IMMUNE | [18174230](http://www.ncbi.nlm.nih.gov/pubmed/18174230?) |
| [SLC8A1](http://geneticassociationdb.nih.gov/cgi-bin/tableview.cgi?table=diseaseview&cond=gene='SLC8A1) | [6546](http://www.ncbi.nlm.nih.gov/sites/entrez?db=gene&cmd=Retrieve&dopt=full_report&list_uids=6546) | Hyperparathyroidism, Secondary | METABOLIC | [20424473](http://www.ncbi.nlm.nih.gov/pubmed/20424473?) |
| [SLC8A1](http://geneticassociationdb.nih.gov/cgi-bin/tableview.cgi?table=diseaseview&cond=gene='SLC8A1) | [6546](http://www.ncbi.nlm.nih.gov/sites/entrez?db=gene&cmd=Retrieve&dopt=full_report&list_uids=6546) | Tobacco Use Disorder | CHEMDEPENDENCY | [20379614](http://www.ncbi.nlm.nih.gov/pubmed/20379614?) |
| [SLC8A1](http://geneticassociationdb.nih.gov/cgi-bin/tableview.cgi?table=diseaseview&cond=gene='SLC8A1) | [6546](http://www.ncbi.nlm.nih.gov/sites/entrez?db=gene&cmd=Retrieve&dopt=full_report&list_uids=6546) | Cardiovascular Diseases | CARDIOVASCULAR | [20109173](http://www.ncbi.nlm.nih.gov/pubmed/20109173?) |
| [RSRC1](http://geneticassociationdb.nih.gov/cgi-bin/tableview.cgi?table=diseaseview&cond=gene='RSRC1) | [51319](http://www.ncbi.nlm.nih.gov/sites/entrez?db=gene&cmd=Retrieve&dopt=full_report&list_uids=51319) | schizophrenia | PSYCH | [19065146](http://www.ncbi.nlm.nih.gov/pubmed/19065146?) |
| [RSRC1](http://geneticassociationdb.nih.gov/cgi-bin/tableview.cgi?table=diseaseview&cond=gene='RSRC1) | [51319](http://www.ncbi.nlm.nih.gov/sites/entrez?db=gene&cmd=Retrieve&dopt=full_report&list_uids=51319) | Type 2 Diabetes\| edema \| rosiglitazone | PHARMACOGENOMIC | [20628086](http://www.ncbi.nlm.nih.gov/pubmed/20628086?) |
| [RSRC1](http://geneticassociationdb.nih.gov/cgi-bin/tableview.cgi?table=diseaseview&cond=gene='RSRC1) | [51319](http://www.ncbi.nlm.nih.gov/sites/entrez?db=gene&cmd=Retrieve&dopt=full_report&list_uids=51319) | Tobacco Use Disorder | CHEMDEPENDENCY | [20379614](http://www.ncbi.nlm.nih.gov/pubmed/20379614?) |
