# Supplementary material for: A Prostate Cancer Model Build by a Novel SVM-ID3 Hybrid Feature Selection Method Using Both Genotyping and Phenotype Data from dbGaP
Source: PLoS One. 2014 Mar 20;9(3):e91404. doi: 10.1371/journal.pone.0091404 (PMC3961262; doi:10.1371/journal.pone.0091404)
Supplement: Table S2 — Whole list of RegulomeDB results. (DOCX) [file pone.0091404.s002.docx]

## Table S2

| rsid | hits | score |
| --- | --- | --- |
| rs1433369 | Motifs\|Footprinting\|IRF, Motifs\|PWM\|DMRT5, Motifs\|Footprinting\|DMRT5, Motifs\|Footprinting\|STAT1, Motifs\|PWM\|IRF, Motifs\|PWM\|STAT1, Chromatin_Structure\|FAIRE, Chromatin_Structure\|DNase-seq, Protein_Binding\|ChIP-seq\|SMARCB1, Protein_Binding\|ChIP-seq\|POLR2A | 2,2 |
| rs11790106 | Motifs\|Footprinting\|Pax-6, Motifs\|PWM\|Pax-6, Chromatin_Structure\|FAIRE, Chromatin_Structure\|DNase-seq, Protein_Binding\|ChIP-seq\|GATA1, Protein_Binding\|ChIP-seq\|HNF4A, Protein_Binding\|ChIP-seq\|HEY1, Protein_Binding\|ChIP-seq\|EP300, Protein_Binding\|ChIP-seq\|SMARCC2, Protein_Binding\|ChIP-seq\|CEBPB, Protein_Binding\|ChIP-seq\|FOXA2, Protein_Binding\|ChIP-seq\|NR3C1, Protein_Binding\|ChIP-seq\|STAT3, Protein_Binding\|ChIP-seq\|POLR2A, Protein_Binding\|ChIP-seq\|FOXA1, Protein_Binding\|ChIP-seq\|SRF, Protein_Binding\|ChIP-seq\|CDX2 | 2,2 |
| rs6774902 | Motifs\|PWM\|MAF, Motifs\|PWM\|c-Ets-1, Motifs\|Footprinting\|c-Ets-1, Motifs\|Footprinting\|MAF, Chromatin_Structure\|DNase-seq, Protein_Binding\|ChIP-seq\|RAD21, Protein_Binding\|ChIP-seq\|CTCF | 2,2 |
| rs17701543 | Motifs\|PWM\|CP2, Chromatin_Structure\|DNase-seq, Protein_Binding\|ChIP-seq\|CTCF | 3,1 |
| rs12644498 | Motifs\|PWM\|REST, Chromatin_Structure\|FAIRE, Chromatin_Structure\|DNase-seq, Protein_Binding\|ChIP-seq\|USF1 | 3,1 |
| rs17375010 | Chromatin_Structure\|DNase-seq, Protein_Binding\|ChIP-seq\|CTCF | 4 |
| rs10788555 | Chromatin_Structure\|FAIRE, Chromatin_Structure\|DNase-seq, Protein_Binding\|ChIP-seq\|STAT1, Protein_Binding\|ChIP-seq\|STAT3 | 4 |
| rs6887293 | Chromatin_Structure\|FAIRE, Chromatin_Structure\|DNase-seq, Protein_Binding\|ChIP-seq\|FOXA1, Protein_Binding\|ChIP-seq\|GATA3 | 4 |
| rs744346 | Chromatin_Structure\|FAIRE, Chromatin_Structure\|DNase-seq, Protein_Binding\|ChIP-seq\|ELK4 | 4 |
| rs4562278 | Chromatin_Structure\|FAIRE, Chromatin_Structure\|DNase-seq, Protein_Binding\|ChIP-seq\|HNF4A | 4 |
| rs10745253 | Motifs\|PWM\|Sox4, Chromatin_Structure\|DNase-seq | 5 |
| rs2296370 | Motifs\|PWM\|NF-1, Protein_Binding\|ChIP-seq\|SMARCA4 | 5 |
| rs2120806 | Motifs\|PWM\|Roaz, Motifs\|Footprinting\|Roaz, Chromatin_Structure\|DNase-seq | 5 |
| rs17001078 | Chromatin_Structure\|FAIRE, Chromatin_Structure\|DNase-seq | 5 |
| rs918285 | Motifs\|PWM\|Msx-3, Chromatin_Structure\|FAIRE, Chromatin_Structure\|DNase-seq | 5 |
| rs3812906 | Motifs\|PWM\|ESR2, Motifs\|PWM\|ESR1, Chromatin_Structure\|DNase-seq | 5 |
| rs2666205 | Chromatin_Structure\|DNase-seq | 5 |
| rs12247568 | Chromatin_Structure\|FAIRE, Chromatin_Structure\|DNase-seq | 5 |
| rs504207 | Motifs\|PWM\|Irf4, Protein_Binding\|ChIP-seq\|SPI1 | 5 |
| rs6708126 | Motifs\|PWM\|MAF, Motifs\|PWM\|AML, Motifs\|Footprinting\|AML, Motifs\|Footprinting\|MAF, Chromatin_Structure\|FAIRE, Chromatin_Structure\|DNase-seq | 5 |
| rs2853668 | Chromatin_Structure\|FAIRE, Chromatin_Structure\|DNase-seq | 5 |
| rs17799219 | Chromatin_Structure\|DNase-seq | 5 |
| rs197265 | Chromatin_Structure\|DNase-seq | 5 |
| rs17363393 | Chromatin_Structure\|FAIRE, Chromatin_Structure\|DNase-seq | 5 |
| rs280986 | Motifs\|PWM\|FOXP1, Chromatin_Structure\|DNase-seq | 5 |
| rs6475584 | Motifs\|PWM\|Sox1, Motifs\|PWM\|Sox4, Motifs\|PWM\|RFX1, Motifs\|PWM\|Sox11, Chromatin_Structure\|DNase-seq | 5 |
| rs4793790 | Chromatin_Structure\|DNase-seq | 5 |
| rs2115101 | Chromatin_Structure\|DNase-seq | 5 |
| rs517036 | Chromatin_Structure\|DNase-seq | 5 |
| rs10106027 | Chromatin_Structure\|DNase-seq | 5 |
| rs3760903 | Chromatin_Structure\|DNase-seq | 5 |
| rs7584223 | Chromatin_Structure\|DNase-seq | 5 |
| rs11729739 | Motifs\|PWM\|Sox13 | 6 |
| rs964130 | Motifs\|PWM\|Sox30 | 6 |
| rs4908656 | Motifs\|PWM\|IPF1, Chromatin_Structure\|FAIRE | 6 |
| rs12980509 | Motifs\|PWM\|HNF1, Motifs\|PWM\|HNF1B | 6 |
| rs7843255 | Motifs\|PWM\|Tcf3 | 6 |
| rs7067548 | Motifs\|PWM\|GCNF | 6 |
| rs2442602 | Motifs\|PWM\|Mafb, Motifs\|PWM\|RSRFC4 | 6 |
| rs3093679 | Motifs\|PWM\|NeuroD | 6 |
| rs7010457 | Motifs\|PWM\|Oct-4(POU5F1), Motifs\|Footprinting\|, Chromatin_Structure\|FAIRE | 6 |
| rs10854395 | Motifs\|PWM\|Arid5a | 6 |
| rs524534 | Motifs\|PWM\|Tcf7l2, Motifs\|PWM\|Lef1 | 6 |
| rs2826802 | Motifs\|PWM\|Hic1 | 6 |
| rs11126869 | Motifs\|PWM\|Gfi-1 | 6 |
| rs9401290 | Motifs\|PWM\|Gata5, Motifs\|PWM\|Sox8 | 6 |
| rs6779266 | Motifs\|PWM\|Nkx2-1 | 6 |
| rs2948268 | Motifs\|PWM\|Oct-1 | 6 |
| rs6676372 | Motifs\|PWM\|Hoxd3, Motifs\|PWM\|Gsh2, Motifs\|PWM\|Lhx1, Motifs\|PWM\|Vax1, Motifs\|PWM\|Dlx2, Motifs\|PWM\|Zfp105, Motifs\|PWM\|Sox4, Motifs\|PWM\|Sox8, Motifs\|PWM\|GSH2, Motifs\|PWM\|Barhl2, Motifs\|PWM\|HOXD3 | 6 |
| rs2711134 | Motifs\|PWM\|PPARG | 6 |
| rs17595858 | Motifs\|PWM\|STAT1 | 6 |
| rs501700 | Motifs\|PWM\|DMRT2, Motifs\|PWM\|DMRT5, Motifs\|PWM\|DMRT4, Motifs\|PWM\|DMRT3 | 6 |
| rs6747704 | Motifs\|PWM\|Sox1 | 6 |
| rs17152800 | Motifs\|PWM\|Tcf3, Chromatin_Structure\|FAIRE | 6 |
| rs10068915 | Motifs\|PWM\|Zfp691 | 6 |
| rs7152946 | Motifs\|PWM\|Sox13 | 6 |
| rs9848588 | No data | 7 |
| rs10195113 | No data | 7 |
| rs12733054 | No data | 7 |
| rs12201462 | No data | 7 |
| rs9462806 | No data | 7 |
| rs1974562 | No data | 7 |
| rs10954845 | No data | 7 |
| rs6997228 | No data | 7 |
| rs2194505 | No data | 7 |
| rs10517581 | No data | 7 |
| rs2103869 | No data | 7 |
| rs7034430 | No data | 7 |
| rs9347691 | No data | 7 |
| rs6851444 | No data | 7 |
| rs11086671 | No data | 7 |
| rs6549458 | No data | 7 |
| rs11944117 | No data | 7 |
| rs6686571 | No data | 7 |
| rs12119983 | No data | 7 |
| rs7183502 | No data | 7 |
| rs13011951 | No data | 7 |
| rs12266639 | No data | 7 |
| rs7024840 | No data | 7 |
| rs7775829 | No data | 7 |
| rs17284653 | No data | 7 |
| rs1379015 | No data | 7 |
| rs1965340 | No data | 7 |
| rs6704731 | No data | 7 |
| rs7876199 | No data | 7 |
| rs17673975 | No data | 7 |
| rs9963110 | No data | 7 |
| rs960278 | No data | 7 |
| rs2602296 | No data | 7 |
| rs17400029 | No data | 7 |
| rs11685549 | No data | 7 |
| rs4517938 | No data | 7 |
| rs7562894 | No data | 7 |
| rs5972169 | No data | 7 |
| rs4782945 | No data | 7 |
| rs12243805 | No data | 7 |
| rs1454186 | No data | 7 |
| rs4827384 | No data | 7 |
| rs11221701 | No data | 7 |
| rs17432165 | No data | 7 |
| rs1470494 | No data | 7 |
| rs1020235 | No data | 7 |
| rs17178580 | No data | 7 |
| rs17111584 | No data | 7 |
| rs11584032 | No data | 7 |
| rs11885120 | No data | 7 |
| rs340542 | No data | 7 |
