# Supplementary material for: A Prostate Cancer Model Build by a Novel SVM-ID3 Hybrid Feature Selection Method Using Both Genotyping and Phenotype Data from dbGaP
Source: PLoS One. 2014 Mar 20;9(3):e91404. doi: 10.1371/journal.pone.0091404 (PMC3961262; doi:10.1371/journal.pone.0091404)
Supplement: Tree S1 — Text representation of tree structure. The tree structure of the SVM-ID3 hybrid model. (DOCX) [file pone.0091404.s003.docx]

## Tree S1

ethni = B

| bmi_cat = aa

| | rs11729739 = ee: 1

| | rs11729739 = ff: 2

| bmi_cat = bb

| | rs17701543 = dd: 1

| | rs17701543 = kk

| | | rs9848588 = dd: 1

| | | rs9848588 = kk

| | | | rs964130 = aa

| | | | | rs10195113 = dd: 1

| | | | | rs10195113 = kk

| | | | | | rs1433369 = ff: 1

| | | | | | rs1433369 = hh

| | | | | | | rs12733054 = ff: 1

| | | | | | | rs12733054 = hh

| | | | | | | | rs17375010 = ff: 1

| | | | | | | | rs17375010 = hh

| | | | | | | | | rs766045 = aa: 2

| | | | | | | | | rs766045 = dd

| | | | | | | | | | ethanol_ca = aa: 2

| | | | | | | | | | ethanol_ca = cc: 1

| | | | rs964130 = dd: 1

| bmi_cat = cc

| | packyrs_ca = aa

| | | rs12201462 = ff: 1

| | | rs12201462 = hh

| | | | rs4908656 = aa

| | | | | rs9462806 = aa

| | | | | | rs1974562 = dd: 1

| | | | | | rs1974562 = kk: 2

| | | | | rs9462806 = dd: 1

| | | | rs4908656 = cc: 1

| | packyrs_ca = bb

| | | rs10954845 = aa

| | | | rs6997228 = aa: 1

| | | | rs6997228 = dd: 2

| | | rs10954845 = dd: 2

| | packyrs_ca = cc

| | | rs10745253 = aa: 2

| | | rs10745253 = dd: 1

| | packyrs_ca = dd

| | | rs12980509 = ff: 1

| | | rs12980509 = hh

| | | | rs2296370 = aa: 1

| | | | rs2296370 = dd: 1

| | | | rs2296370 = kk: 2

| | packyrs_ca = ee

| | | rs7843255 = aa: 1

| | | rs7843255 = dd: 2

| | | rs7843255 = kk: 1

| | packyrs_ca = ff

| | | rs2194505 = ee: 2

| | | rs2194505 = ff: 1

| | packyrs_ca = xx

| | | rs2120806 = ff: 1

| | | rs2120806 = hh: 2

| bmi_cat = dd

| | rs10517581 = aa

| | | rs2103869 = dd: 2

| | | rs2103869 = kk

| | | | rs10788555 = gg: 2

| | | | rs10788555 = kk

| | | | | rs7067548 = aa

| | | | | | rs17001078 = ee

| | | | | | | rs918285 = ee: 1

| | | | | | | rs918285 = xx: 2

| | | | | | rs17001078 = ff: 2

| | | | | rs7067548 = cc: 2

| | rs10517581 = dd

| | | rs9462806 = aa: 2

| | | rs9462806 = dd: 1

| bmi_cat = xx

| | rs7034430 = ff: 2

| | rs7034430 = hh: 1

ethni = J

| bmi_cat = aa

| | rs2442602 = aa: 1

| | rs2442602 = dd

| | | rs3093679 = dd: 2

| | | rs3093679 = kk: 1

| | rs2442602 = kk: 2

| bmi_cat = bb

| | rs9347691 = aa

| | | rs6851444 = aa

| | | | rs11086671 = aa

| | | | | rs6887293 = dd: 2

| | | | | rs6887293 = kk

| | | | | | rs3812906 = dd: 2

| | | | | | rs3812906 = kk

| | | | | | | rs6549458 = aa

| | | | | | | | rs11944117 = aa: 1

| | | | | | | | rs11944117 = dd: 2

| | | | | | | rs6549458 = cc: 2

| | | | rs11086671 = dd: 2

| | | rs6851444 = dd: 2

| | rs9347691 = dd

| | | rs2666205 = aa: 2

| | | rs2666205 = cc: 1

| bmi_cat = cc

| | rs7010457 = aa

| | | rs10854395 = aa

| | | | rs12644498 = aa: 1

| | | | rs12644498 = dd: 2

| | | | rs12644498 = kk

| | | | | rs12247568 = aa

| | | | | | rs6686571 = aa

| | | | | | | rs7843255 = dd: 1

| | | | | | | rs7843255 = kk

| | | | | | | | rs504207 = aa: 2

| | | | | | | | rs504207 = dd

| | | | | | | | | rs12119983 = aa: 2

| | | | | | | | | rs12119983 = cc: 1

| | | | | | | | rs504207 = kk: 1

| | | | | | rs6686571 = cc

| | | | | | | rs6708126 = aa: 1

| | | | | | | rs6708126 = dd: 2

| | | | | rs12247568 = dd: 1

| | | rs10854395 = dd

| | | | rs2853668 = cc: 2

| | | | rs2853668 = hh

| | | | | rs524534 = aa: 1

| | | | | rs524534 = dd: 2

| | | | | rs524534 = kk: 2

| | | rs10854395 = kk: 2

| | rs7010457 = dd: 1

| bmi_cat = dd: 2

ethni = L

| bmi_cat = aa

| | rs17799219 = dd: 2

| | rs17799219 = kk

| | | rs7183502 = aa

| | | | rs13011951 = aa

| | | | | rs12266639 = dd: 2

| | | | | rs12266639 = kk

| | | | | | rs2826802 = dd: 2

| | | | | | rs2826802 = kk

| | | | | | | rs7024840 = aa: 1

| | | | | | | rs7024840 = hh: 2

| | | | rs13011951 = dd: 2

| | | rs7183502 = dd: 2

| bmi_cat = bb

| | rs197265 = aa

| | | rs17363393 = dd: 1

| | | rs17363393 = kk

| | | | rs280986 = dd: 2

| | | | rs280986 = kk

| | | | | rs11790106 = dd: 2

| | | | | rs11790106 = kk

| | | | | | rs11126869 = dd: 2

| | | | | | rs11126869 = kk

| | | | | | | fh_prca = aa

| | | | | | | | pa_cat = cc: 2

| | | | | | | | pa_cat = dd: 1

| | | | | | | fh_prca = jj: 2

| | | | | | | fh_prca = ww

| | | | | | | | rs7775829 = cc: 2

| | | | | | | | rs7775829 = hh

| | | | | | | | | rs9401290 = aa

| | | | | | | | | | rs17284653 = dd: 2

| | | | | | | | | | rs17284653 = kk

| | | | | | | | | | | MitoG752A = kk

| | | | | | | | | | | | rs1379015 = dd: 2

| | | | | | | | | | | | rs1379015 = kk

| | | | | | | | | | | | | rs1965340 = aa: 1

| | | | | | | | | | | | | rs1965340 = dd: 2

| | | | | | | | | | | MitoG752A = xx: 2

| | | | | | | | | rs9401290 = dd

| | | | | | | | | | rs6704731 = aa: 2

| | | | | | | | | | rs6704731 = dd: 1

| | | | | | | | | rs9401290 = kk: 1

| | rs197265 = dd: 1

| | rs197265 = kk: 2

| | rs197265 = xx: 1

| bmi_cat = cc

| | fh_prca = aa

| | | rs6475584 = aa: 1

| | | rs6475584 = dd: 2

| | | rs6475584 = kk: 1

| | fh_prca = jj

| | | rs7876199 = aa: 1

| | | rs7876199 = kk

| | | | rs17673975 = aa

| | | | | rs6779266 = cc: 1

| | | | | rs6779266 = hh

| | | | | | rs7024840 = aa

| | | | | | | rs16863955 = aa: 2

| | | | | | | rs16863955 = dd: 1

| | | | | | rs7024840 = cc: 1

| | | | rs17673975 = dd: 1

| | fh_prca = ww

| | | pa_cat = aa

| | | | rs9963110 = cc: 2

| | | | rs9963110 = hh

| | | | | rs960278 = aa

| | | | | | rs2115101 = aa

| | | | | | | rs2602296 = aa

| | | | | | | | rs17400029 = dd: 2 {2=1, 1=0}

| | | | | | | | rs17400029 = kk

| | | | | | | | | rs2948268 = aa

| | | | | | | | | | rs11685549 = dd: 2 {2=1, 1=0}

| | | | | | | | | | rs11685549 = kk

| | | | | | | | | | | rs6676372 = dd

| | | | | | | | | | | | rs4793790 = aa: 1

| | | | | | | | | | | | rs4793790 = dd: 2

| | | | | | | | | | | rs6676372 = kk: 1

| | | | | | | | | rs2948268 = dd: 2

| | | | | | | rs2602296 = dd: 2

| | | | | | rs2115101 = dd

| | | | | | | eversmoke = aa: 2

| | | | | | | eversmoke = ww: 1

| | | | | rs960278 = dd: 2

| | | pa_cat = bb

| | | | rs2711134 = aa

| | | | | rs4517938 = dd: 1

| | | | | rs4517938 = kk

| | | | | | rs517036 = aa

| | | | | | | rs7843255 = dd: 1

| | | | | | | rs7843255 = kk

| | | | | | | | rs7562894 = dd: 1

| | | | | | | | rs7562894 = kk

| | | | | | | | | rs17595858 = dd: 1

| | | | | | | | | rs17595858 = kk

| | | | | | | | | | rs5972169 = aa: 1

| | | | | | | | | | rs5972169 = hh

| | | | | | | | | | | rs4782945 = cc: 1

| | | | | | | | | | | rs4782945 = hh

| | | | | | | | | | | | rs12243805 = dd: 1

| | | | | | | | | | | | rs12243805 = kk

| | | | | | | | | | | | | rs1454186 = aa: 1

| | | | | | | | | | | | | rs1454186 = kk

| | | | | | | | | | | | | | rs4827384 = aa

| | | | | | | | | | | | | | | rs11221701 = aa

| | | | | | | | | | | | | | | | rs501700 = aa: 2

| | | | | | | | | | | | | | | | rs501700 = dd: 1}

| | | | | | | | | | | | | | | | rs501700 = kk: 2

| | | | | | | | | | | | | | | rs11221701 = dd: 1

| | | | | | | | | | | | | | rs4827384 = kk: 1

| | | | | | rs517036 = dd: 1

| | | | | | rs517036 = kk: 1

| | | | rs2711134 = dd: 2

| | | pa_cat = cc

| | | | currsmoke = aa: 2

| | | | currsmoke = ww

| | | | | rs6686571 = aa

| | | | | | rs17432165 = aa: 1

| | | | | | rs17432165 = dd: 2

| | | | | rs6686571 = cc: 2

| | | | | rs6686571 = hh: 2

| | | pa_cat = dd

| | | | rs1470494 = aa

| | | | | rs744346 = aa

| | | | | | rs12644498 = dd: 1

| | | | | | rs12644498 = kk

| | | | | | | rs6774902 = dd: 1

| | | | | | | rs6774902 = kk

| | | | | | | | rs6747704 = dd: 1

| | | | | | | | rs6747704 = kk

| | | | | | | | | rs17152800 = dd: 1

| | | | | | | | | rs17152800 = kk: 2

| | | | | rs744346 = cc: 1

| | | | rs1470494 = cc

| | | | | rs6549458 = aa: 1

| | | | | rs6549458 = hh: 2

| | | pa_cat = ee

| | | | rs10068915 = aa: 2

| | | | rs10068915 = dd: 1

| | | | rs10068915 = kk

| | | | | rs1020235 = aa

| | | | | | rs10106027 = dd: 1

| | | | | | rs10106027 = kk

| | | | | | | rs17111584 = aa

| | | | | | | | rs17178580 = aa: 2

| | | | | | | | rs17178580 = cc: 1

| | | | | | | rs17111584 = dd: 1

| | | | | rs1020235 = dd: 1

| | | pa_cat = xx

| | | | ethanol_ca = aa: 2

| | | | ethanol_ca = bb: 1

| bmi_cat = dd

| | d_lyco_cat = aa

| | | eversmoke = aa: 1

| | | eversmoke = ww: 2

| | d_lyco_cat = bb

| | | rs3760903 = dd

| | | | rs12266639 = dd: 2

| | | | rs12266639 = kk

| | | | | rs11584032 = dd: 1

| | | | | rs11584032 = xx: 2

| | | rs3760903 = kk: 2

| | d_lyco_cat = cc

| | | rs4562278 = aa: 1

| | | rs4562278 = cc

| | | | fh_prca = aa: 1

| | | | fh_prca = ww: 2

| | d_lyco_cat = dd

| | | rs17363393 = dd: 1

| | | rs17363393 = kk

| | | | rs11885120 = dd: 1

| | | | rs11885120 = kk

| | | | | rs6779266 = cc: 1

| | | | | rs6779266 = hh: 2

| | d_lyco_cat = ee

| | | rs7584223 = aa

| | | | rs7152946 = aa

| | | | | rs340542 = aa: 2

| | | | | rs340542 = dd

| | | | | | pa_cat = bb: 1

| | | | | | pa_cat = cc: 2

| | | | rs7152946 = cc: 1

| | | rs7584223 = dd: 1

| | d_lyco_cat = xx: 2
